# Optimizing Nanopore Sequencing for Rapid Detection of Microbial Species and Antimicrobial Resistance in Patients at Risk of Surgical Site Infections

**DOI:** 10.1128/msphere.00964-21

**Published:** 2022-02-16

**Authors:** Emma Whittle, Jennifer A. Yonkus, Patricio Jeraldo, Roberto Alva-Ruiz, Heidi Nelson, Michael L. Kendrick, Thomas E. Grys, Robin Patel, Mark J. Truty, Nicholas Chia

**Affiliations:** a Division of Surgical Research, Department of Surgery, Mayo Clinic, Rochester, Minnesota, USA; b Division of Hepatobiliary & Pancreatic Surgery, Department of Surgery, Mayo Clinic, Rochester, Minnesota, USA; c Division of Research and Optimal Patient Care, Cancer Programs, American College of Surgeons, Chicago, Illinois, USA; d Department of Laboratory Medicine and Pathology, Mayo Clinic, Phoenix, Arizona, USA; e Division of Clinical Microbiology, Department of Laboratory Medicine and Pathology, Mayo Clinic, Rochester, Minnesota, USA; Hackensack Meridian Health Center for Discovery and Innovation

**Keywords:** antimicrobial resistance, microbial detection, nanopore sequencing, surgical site infection

## Abstract

Surgical site infections (SSI) are a significant burden to patients and health care systems. We evaluated the use of Nanopore sequencing (NS) to rapidly detect microbial species and antimicrobial resistance (AMR) genes present in intraoperative bile aspirates. Bile aspirates from 42 patients undergoing pancreatic head resection were included. Three methods of DNA extraction using mechanical cell lysis or protease cell lysis were compared to determine the optimum method of DNA extraction. The impact of host DNA depletion, sequence run duration, and use of different AMR gene databases was also assessed. To determine clinical value, NS results were compared to standard culture (SC) results. NS identified microbial species in all culture positive samples. Mechanical lysis improved NS detection of cultured species from 60% to 76%, enabled detection of fungal species, and increased AMR predictions. Host DNA depletion improved detection of streptococcal species and AMR correlation with SC. Selection of AMR database influenced the number of AMR hits and resistance profile of 13 antibiotics. AMR prediction using CARD and ResFinder 4.1 correctly predicted 79% and 81% of the bile antibiogram, respectively. Sequence run duration positively correlated with detection of AMR genes. A minimum of 6 h was required to characterize the biliary microbes, resulting in a turnaround time of 14 h. Rapid identification of microbial species and AMR genes can be achieved by NS. NS results correlated with SC, suggesting that NS may be useful in guiding early antimicrobial therapy postsurgery.

**IMPORTANCE** Surgical site infections (SSI) are a significant burden to patients and health care systems. They increase mortality rates, length of hospital stays, and associated health care costs. To reduce the risk of SSI, surgical patients are administered broad-spectrum antibiotics that are later adapted to target microbial species detected at the site of surgical incision. Use of broad-spectrum antibiotics can be harmful to the patient. We wanted to develop a rapid method of detecting microbial species and their antimicrobial resistance phenotypes. We developed a method of detecting microbial species and predicting resistance phenotypes using Nanopore sequencing. Results generated using Nanopore sequencing were similar to current methods of detection but were obtained in a significantly shorter amount of time. This suggests that Nanopore sequencing could be used to tailor antibiotics in surgical patients and reduce use of broad-spectrum antibiotics.

## INTRODUCTION

Surgical site infections (SSI) are the most common and costly of hospital-acquired infections within the U.S., accounting for 20% of all hospital-acquired infections (1). Ranging from superficial skin infections to life-threatening sepsis (2), SSI increase hospital stay by an average of 9.7 days and account for a 2- to 11-fold increase in risk of mortality (1). The incidence rate of SSI within the U.S. is estimated at 2–5% of surgical patients (1–4), accounting for an estimated 160,000 to 300,000 cases annually and costing the U.S. health care an estimated $3.5 to $10 billion each year (1).

The incidence rate of SSI varies across surgical procedures, specialties, and hospitals, and has been reported to vary from 0.1% to 50% (2). Pancreaticoduodenectomy, commonly referred to as the Whipple procedure, is a major surgical procedure typically performed to remove cancerous tumors from the pancreas and is associated with particularly high rates of SSI. Incidence rates range from 25% to 45% of patients undergoing the procedure (5, 6), and the occurrence of an SSI increases rates of mortality and the need for additional invasive procedures, and can result in delay or failure to complete adjuvant chemotherapy (7, 8).

An estimated 60% of SSIs are preventable (9). With regard to preventing SSIs in pancreaticoduodenectomy patients, this institution developed a standardized broad-spectrum prophylactic antimicrobial regimen for all patients undergoing pancreaticoduodenectomy. The regimen was based on microbial species and AMR phenotypes detected in historical SSI culture data and includes a 5-day course of intravenously administered ceftriaxone and metronidazole (10, 11). Antimicrobial therapy may then be further optimized following identification of microbial species and AMR typing from intraoperative bile cultures.

Rapid etiologic diagnosis of microbial biliary contamination can facilitate timely and rational postoperative antimicrobial therapy, reducing the risk of SSI developing. Standard cultures (SC), however, can take days to weeks to return actionable results (12, 13) and are typically received over the course of 2–4 days. Bile cultures are often polymicrobial, a mixture of anaerobic and aerobic species, carry multiple antimicrobial resistance (AMR) phenotypes, and contain fungal species (11, 14–16). This contributes to delays in timely results, and the temporal separation between the initial broad-spectrum treatment and the acquisition of all the diagnostic information often results in uncertainty and the administration of multiple empirical antimicrobials (17). Moreover, an estimated 30% of SSIs are associated with negative culture results (18), and bile cultures have been found to be poor predictors of AMR patterns in postoperative infections (16). Extensive use of broad-spectrum antimicrobials contributes toward the emergence of AMR in pathogens (19, 20). Furthermore, unneeded antimicrobials can result in harmful side effects (21) with no benefit to the patient, and attempts to target subpopulations for prophylactic antibiotic therapy based on other clinical indicators have failed (22).

Metagenomic-based sequencing approaches can directly quantify microbes and AMR genes and have the potential to overcome the limitations associated with the standard practices of culture by combining speed with comprehensive coverage of all microbes present (23). Specifically, metagenomics enables simultaneous detection of all bacteria, fungi, viruses, and protozoa, without the requirement for specifically designed primers (24), and can be used to detect complex multilocus pathogenic traits, such as AMR (24, 25). Next-generation sequencing platforms, such as Illumina and ION Torrent, have been widely used for metagenomic sequencing, including previous characterization of the bile microbiome in healthy and diseased states (26, 27). However, in practice, these technologies require at least 16 h for the sequencing run alone, and analysis of the sequencing data can only be performed once the sequence run has concluded (24, 28). When considering sample preparation and analysis, this leads to an overall sample-to-result turnaround time greater than 24 h. Recently, there has been growing interest in metagenomic Nanopore sequencing (NS) for rapid microbial identification and AMR genotyping (17, 28–45). NS involves use of protein nanoparticles set in electrically-resistant polymer membranes contained within devices referred to as flow cells (46, 47). Single DNA or RNA molecules are driven through individual nanopores using electrophoresis, with the passing of each nucleotide detected as a temporary shift in the ionic current (46, 47). Changes to the electrical current are used to determine nucleotide base sequence (base calling) (46, 47), enabling real-time sequence analysis and detection of microbial DNA/RNA to be performed. This has reduced overall sample-to-answer time to as little as 6 h (17, 28, 38), and clinical application of the technology has been demonstrated in several proof-of-concept studies that highlight its ability to detect and characterize microbes from a wide array of sample types (28, 38–45), to monitor AMR in the hospital setting (29–32) and carry out real-time surveillance of viral outbreaks (33–37).

Here, we examine the use of NS technology to provide a rapid, comprehensive, and accurate profile of microbial pathogens and AMR from the polymicrobial environment of bile. More specifically, we compare the results of NS using different methods of DNA extraction, library preparation, and analysis protocols with SC testing from bile and SSI sites as a companion work on NS’s ability to predict clinical outcomes (48). We quantify our results in terms of overlap with patient outcomes, identifying best NS practices for identifying potential pathogens and AMR genes. Interestingly, our data identify causal SSI organisms that would not be treated by the preemptive antibiotics used at our institution, because of associated AMR genes in noncultured bacteria. Collectively, this raises the possibility that NS screening of intraoperative bile samples can not only rapidly identify patients at risk of SSI, but also enable targeted antimicrobial therapy within 24 h of surgery that potentially could have prevented SSIs. This work provides data and testing for future development of a clinical diagnostic tool with the potential for reducing SSIs and improving antibiotic stewardship with benefits to both patients and health care providers.

## RESULTS

### Detection of microbial DNA in bile aspirates.

Biliary microbes were identified in 54.7% (23/42) of patients using SC techniques. This included bacteria in 35.7% (15/42) of patients, fungi in 2.4% (1/42) of patients, and both bacteria and fungi in 16.7% (7/42) of patients. NS identified 100% of cases with biliary microbes within 14 h of sample collection compared to the average time of 98 h (IQR 80–152 h) it took SC. Additionally, in two cases of biliary fungi, species identification using SC was not achieved while NS identified down to the species level. NS also identified fungal species in two cases where fungal cultures were negative, indicating improved detection of fungi in bile. Method of DNA extraction and depletion of host DNA influenced the amount of DNA extracted from the bile samples (Fig. S1 in the supplemental material) and the number of bacterial reads sequenced (Table 1).

**TABLE 1:** Average number of sequenced and classified reads generated from intraoperative bile aspirates using nanopore sequencing

Kit	No. of classified reads	Human		Bacterial		Fungal	
		Reads	Abundance	Reads	Abundance	Reads	Abundance
Phenol Chloroform	841,011	668,730	78.09	135,702	21.85	140	0.03
Phenol Chloroform plus NEBNext	975,567	657,852	66.45	275,274	33.46	291	0.04
QIAamp Blood	935,444	901,412	97.24	34,031	2.76	1.0	0.00
QIAamp Blood plus NEBNext	710,251	564,224	78.30	146,014	21.58	4	0.00
Powersoil Pro	798,541	703,896	86.22	94,640	13.71	143	0.03
*P* values	0.3610	0.2200	0.0132	0.0159	0.0158	0.1800	0.4160

Analysis of the negative controls detected very low levels of microbial DNA, with an average number of 8.14 bacterial reads identified in the phenol chloroform negative controls (range = 1–44 reads), 12.35 reads detected in the QIAamp Blood negative controls (range = 1–111 reads), 14.37 detected in the Powersoil Pro negative controls (range = 1–71 reads), and 14.53 in the NEBNext microbiome enrichment negative controls (range = 1–71 reads) (Fig. S2). This indicated that very little microbial contamination occurred during DNA extraction and library preparation.

### Identification of bacterial species.

**FIG 1:**
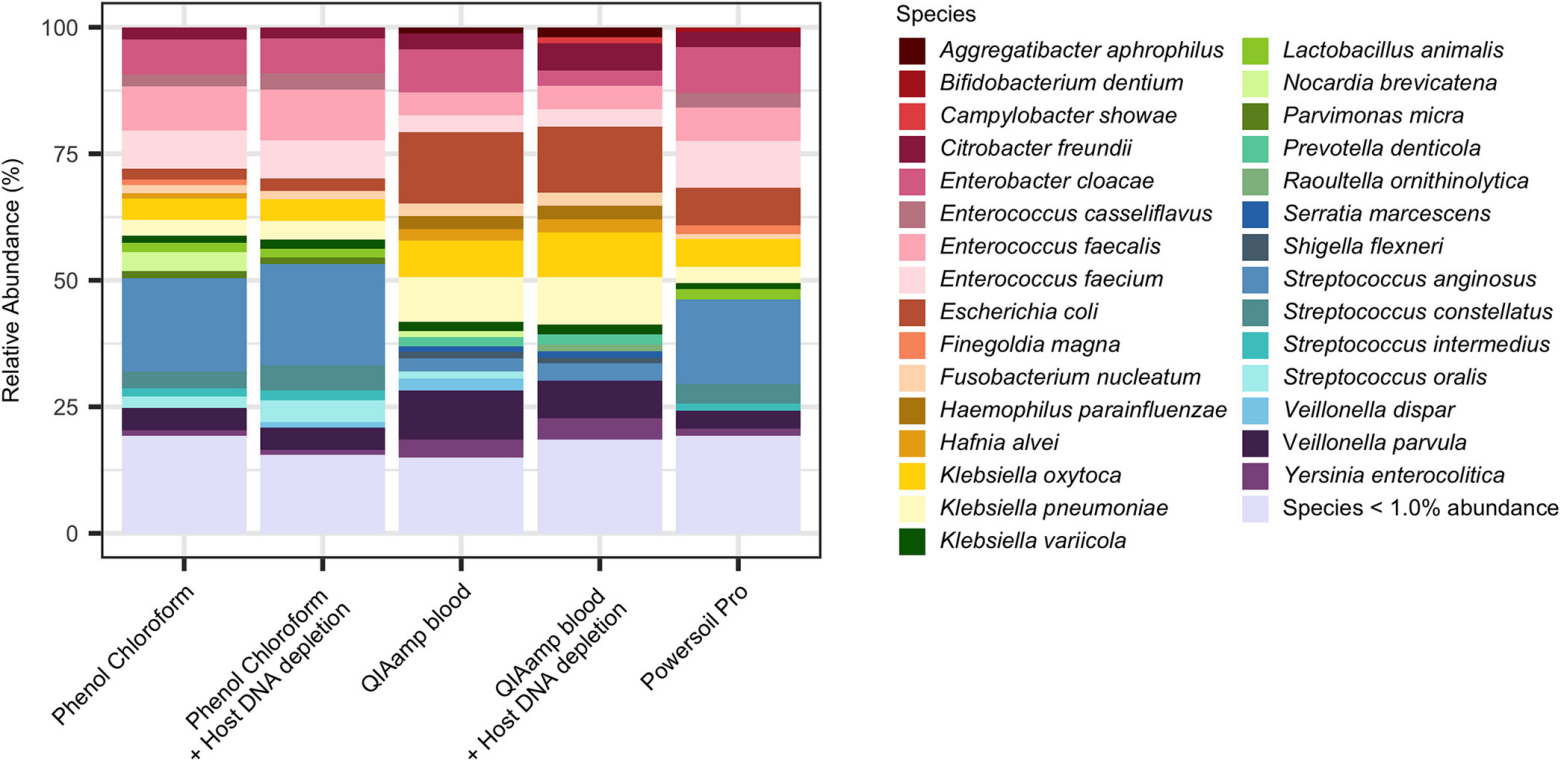
Analysis of microbial species present in bile duct aspirates. Relative abundance of dominant bacterial species detected by each DNA extraction approach was determined to identify differences in microbial identification across the different methods of DNA extraction.

Bacterial species detected in the bile using NS were variable and specific to individual patients. In some samples, a single bacterial species dominated (for example, Enterobacter cloacae, Enterococcus faecalis, Enterococcus faecium, or Streptococcus anginosus), while in others, multiple bacterial species were detected at similar abundance levels. Overall, the bile aspirates were dominated by Enterococcus, Klebsiella, and Streptococcus species and, to a lesser extent, Citrobacter freundii, Escherichia coli, E. cloacae, Fusobacterium nucleatum, and Veillonella species. (Fig. 1) (Table S1). Analysis of beta-diversity of the bacterial populations detected revealed that the phenol chloroform DNA extraction method resulted in characterization of a distinctly different bacterial population compared to the two silica-membrane DNA extraction kits (Fig. S3).

Comparison of bacterial species detected using the different protocols revealed that detection of Bifidobacterium, Nocardia, Streptococcus, and Veillonella species was significantly influenced by the method used (Table S1). Direct comparison of the different methods revealed that the QIAamp Blood protocol resulted in reduced detection of Streptococcus oralis (*P* value = 0.0001), Streptococcus intermedius (*P* value = 0.0002), S. anginosus (*P* value = 0.0003), Bifidobacterium breve (*P* value = 0.0029), Streptococcus constellatus (*P* value = 0.0044), and Nocardia brevicatena (*P* value = 0.023) compared to the phenol chloroform method, suggesting that protease cell lysis may be less efficient at extracting DNA from Gram-positive bacteria. When comparing the results generated from the QIAamp Blood samples that underwent host DNA depletion, there were no significant differences, indicating that potential issues with the kit could be resolved by host DNA depletion. In addition to increasing detection of streptococci in QIAamp Blood processed samples, use of host DNA depletion also reduced detection of *N. brevicatena* in both the phenol chloroform processed samples and the QIAamp Blood samples (*P values *= 0.000002 and 0.0000002, respectively). Analysis of the Powersoil Pro samples found no significant differences compared to QIAamp Blood processed samples.

Analysis of the negative controls found that bacterial species detected in the negative controls were different compared to bacterial species detected in bile (Fig. S4). DNA detected in the negative controls was predominately human and, to a lesser extent, Moraxella osloensis, Shigella flexneri, Cutibacterium acnes, and S. anginosus (Fig. S5). Comparison of species detected using NS and SC revealed that on average NS detected 75% of cultured species when the phenol chloroform method was used, 60% when the QIAamp Blood kit was used, and 77% when the Powersoil Pro kit was used. There were no significant differences in the percentage of cultured species detected across the different NS methods used.

### Detection of fungi.

**FIG 2:**
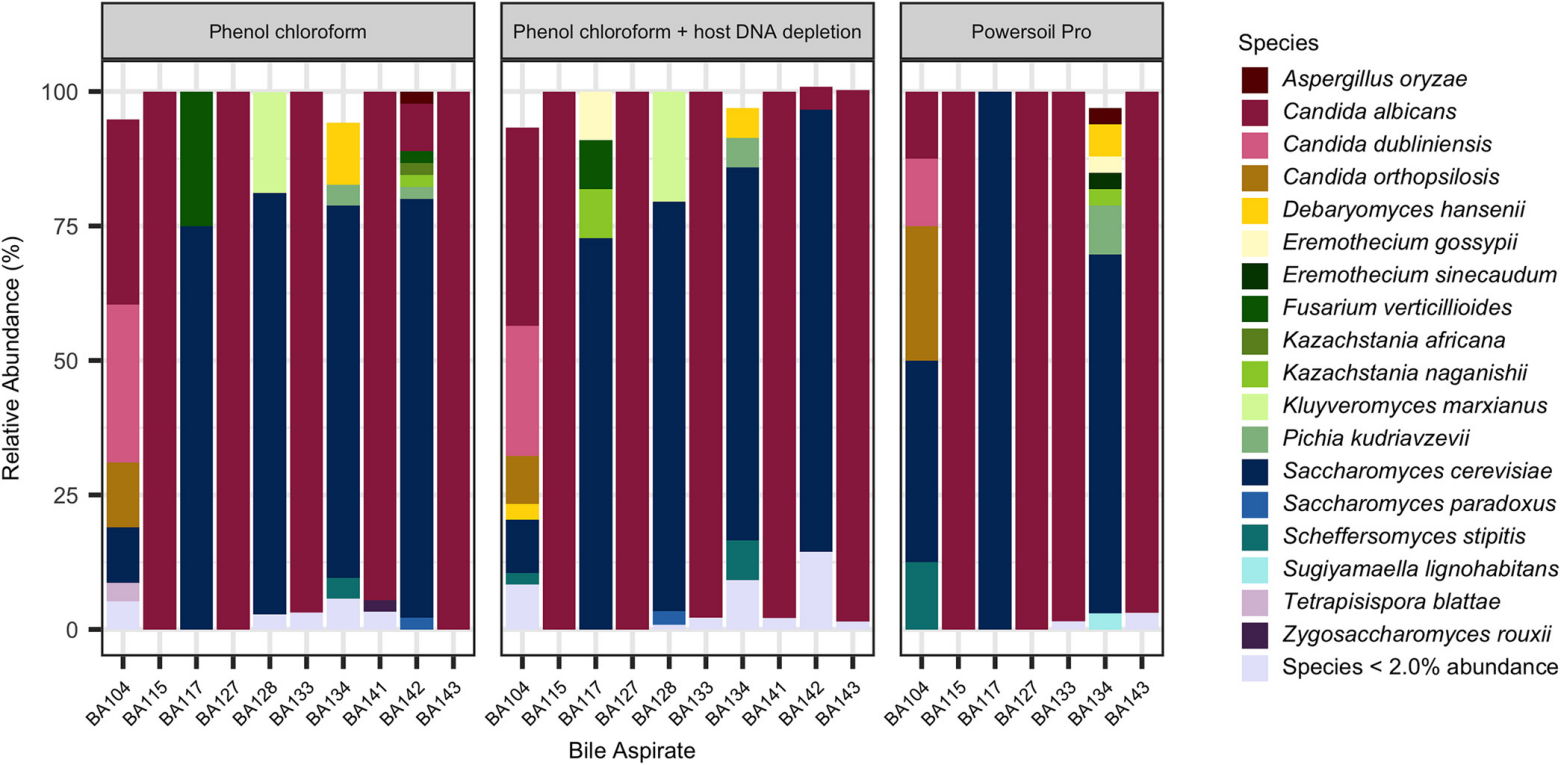
Detection of fungal species from bile duct aspirates collected during pancreaticoduodenectomy surgery. Relative abundance of fungal species detected using nanopore sequencing and the phenol chloroform method, the phenol chloroform method plus host DNA depletion, or the Powersoil Pro method of DNA extraction.

Fungal species were detected in 8 bile aspirates using SC, and in 10 bile aspirates using NS. Comparison of fungal species reported by NS found that the phenol chloroform method detected 76.2% and the Powersoil Pro method detected 72.2% of cultured fungal species. QIAamp Blood samples were not included in this analysis due to insufficient fungal reads, indicating that bead beating lysis is required for successful detection of fungal reads. SC identified fungal species in 75% (6/8) of culture positive samples, and in 83% (5/6) of samples a single fungal species was detected, either *Candida* species or Saccharomyces cerevisiae. In contrast, NS identified fungal species in 100% (10/10) of samples with fungal DNA detected, and while *Candida* species and S. cerevisiae were observed to the dominant species present in the bile aspirates, 50% (5/10) of samples were polymicrobial (Fig. 2). In samples where C. albicans was detected, the sample was typically found to be monoclonal, whereas in samples dominated by S. cerevisiae, additional, low abundant fungal species were detected (Fig. 2). Comparison of fungal species detected using the phenol chloroform method and the Powersoil Pro method revealed that in the polymicrobial samples, there were differences in the levels of low abundant species detected by the two methods (Fig. 2). This suggested that the method of DNA extraction influenced detection of fungal species using NS. Similarly, analysis of fungal species detected using the phenol chloroform with or without host DNA depletion also demonstrated slight differences in the detection rate of the low abundant fungal species. Use of host DNA depletion increased the number of fungal reads sequenced (Table 1), resulting in improved detection of low abundant fungal species. The phenol chloroform processed samples with host DNA depletion yielded the highest level of fungal detection, with over 1,000 fungal reads detected in 3 samples when the method was applied (Bile aspirate (BA)128, BA133, BA141).

### Detection of acquired antimicrobial resistance phenotypes.

SC identified AMR phenotypes in 95.5% of bacterial bile cultures (21/22 samples), and NS predicted AMR phenotypes in 100% (22/22) of bacterial positive samples. On average, NS using the phenol chloroform method predicted 60.7% of resistance phenotypes observed using SC (range = 0–100), NS using the QIAamp Blood protocol predicted 50.9% of observed AMR phenotypes (range = 0–100), and NS using the Powersoil Pro protocol predicted an average of 40.6% AMR phenotypes (range = 0–100). NS sequencing also predicted a higher number of AMR phenotypes than observed by SC (Table 2).

**TABLE 2:** Detection of antimicrobial resistance phenotypes^*a*^

Method	*n* of AMR phenotypes (mean)	No. of samples with AMR Phenotype										
		AMI	CAR	CEPH	Mon	PEN	GLY	LIN	MAC	FLU	TET	STR
Standard culture	0 - 6 (2.4)	2	2	15	1	18	3	1	3	1	0	*na*
Phenol Chloroform	2 - 38 (17.4)	10	0	14	7	14	2	8	12	3	14	12
QIAamp Blood	3 - 30 (10.7)	2	0	15	4	15	1	2	4	4	7	4
Powersoil Pro	3 - 34 (15.1)	8	0	7	2	7	1	3	9	2	8	9

a Antimicrobial resistance (AMR) phenotypes detected in biliary aspirates by standard culture were compared to predicted AMR phenotypes detected by nanopore sequencing. AMI, aminoglycosides; CAR, carbapenems; CEPH, cephalosporins; MON, monobactams; PEN, penicillins; GLY, glycopeptides; LIN, lincosamides; MAC, macrolides; FLU, fluoroquinolones; TET, tetracyclines; STR, streptogramins. *na*, antibiotic class where standard culture susceptibility testing was not performed.

Analysis of the AMR phenotypes predicted by NS found that DNA extraction and use of host DNA depletion significantly influenced AMR predictions. Increased prediction of 16 AMR phenotypes was observed when bead beating was utilized (with the phenol chloroform method or Powersoil Pro method) compared to protease lysis (with the QIAamp Blood method) (Table 3). Predicted resistance for 15 antibiotics was significantly increased using phenol chloroform processed samples compared to the QIAamp Blood processed samples, and 11 were significantly increased using Powersoil Pro processed samples compared to the QIAamp Blood processed samples. (Table 3). There were no significant differences in AMR prediction rates between the phenol chloroform processed samples and the Powersoil Pro processed samples.

**TABLE 3:** Differential prediction of antimicrobial resistance phenotypes using different methods of DNA extraction^*a*^

Antibiotic [class]	QIAamp Blood *vs* phenol chloroform		QIAamp Blood vs Powersoil Pro	
	Fold change	*P* value	Fold change	*P* value
Amikacin [AMI]	*–IFN*	0.0450	*–IFN*	0.1090
Azithromycin [MAC]	*–IFN*	0.0229	*–IFN*	0.0071
Clindamycin [LIN]	–3.82	0.0412	–3.28	0.4376
Dalfopristin [STR]	–5.73	0.0500	–3.94	0.1900
Dibekacin [AMI]	–5.73	0.0500	–10.50	0.0140
Erythromycin [MAC]	–2.86	0.0181	–3.61	0.0216
Gentamicin [AMI]	–5.73	0.0500	*–IFN*	0.0140
Lincomycin [LIN]	–3.82	0.0412	–3.28	0.4376
Netilmicin [AMI]	–5.73	0.0500	–10.50	0.0140
Pristinamycin ia [STR]	–3.50	0.0141	–4.37	0.0213
Pristinamycin iia [STR]	–5.73	0.0500	–3.94	0.1900
Quinupristin [STR]	–3.50	0.0141	–4.37	0.0213
Sisomicin [AMI]	–4.77	0.0970	–9.19	0.0350
Telithromycin [MAC]	–7.64	0.0124	–10.50	0.0138
Tobramycin [AMI]	–6.68	0.0250	–10.50	0.0140
Virginiamycin s [STR]	–3.50	0.0141	–4.37	0.0213

a Predicted antibiotic resistance present in bile aspirates was determined by aligning sequenced reads generated from the phenol chloroform, QIAamp Blood, and Powersoil Pro methods to the ResFinder 4.1 database of acquired antimicrobial resistance (AMR) genes. Detection rates across 22 bile aspirates was determined, and the Wilcoxon test was used to identify antibiotics with altered predicted value across the three methods. –*IFN*, samples where the QIAamp Blood failed to detect resistance; AMI, aminoglycoside; MAC, macrolide; LIN, lincosamide; STR, streptogramin.

Use of host DNA depletion increased the number of AMR phenotypes predicted by NS. Host DNA depletion performed on phenol chloroform processed samples resulted in increased prediction of 9 AMR phenotypes, while prediction of 12 AMR phenotypes was increased in QIAamp Blood processed samples that underwent host DNA depletion (Table 4). Increased AMR predictions following host DNA depletion resulted in improved prediction of resistance phenotypes observed by SC using QIAamp Blood processed samples (50.9 versus 81.1% correlation with SC, *P* value = 0.0269). Comparison of the predictive power of the phenol chloroform protocol with and without host DNA depletion found no significant changes in AMR predictive power. However, use of host DNA depletion increased the total number of predicted AMR phenotypes from an average of 15.8 to 20.8 and improved AMR correlation with SC in 7 samples.

**TABLE 4:** Differential prediction of antimicrobial resistance when host DNA depletion is applied^*a*^

Antibiotic	Phenol chloroform vs phenol chloroform^¶^		QIAamp blood vs QIAamp blood^¶^	
	Fold change	*P* value	Fold change	*P* value
Amoxicillin [PEN]	1.54	0.0167	1.40	0.1116
Amoxicillin + clavulanic acid [PEN]	2.00	0.0380	2.80	0.0068
Ampicillin [PEN]	1.43	0.0342	1.40	0.1116
Ampicillin + clavulanic acid [PEN]	2.00	0.0380	2.80	0.0068
Cefotaxime [CEPH]	1.40	0.2366	2.60	0.0043
Cefoxitin [CEPH]	1.43	0.3658	3.85	0.0039
Ceftazidime [CEPH]	1.40	0.2366	2.80	0.0068
Cephalothin [CEPH]	1.64	0.0290	1.17	0.5820
Clindamycin [LIN]	1.38	0.3740	4.20	0.0344
Lincomycin [LIN]	1.38	0.3740	4.90	0.0131
Piperacillin [PEN]	1.54	0.0167	1.40	0.1116
Piperacillin + tazobactam PEN]	2.00	0.0380	2.80	0.0068
Pristinamycin ia [STR]	1.36	0.2307	3.27	0.0368
Quinupristin [STR]	1.36	0.2307	3.27	0.0368
Ticarcillin [CARB]	1.54	0.0167	1.40	0.1116
Ticarcillin + clavulanic acid [CARB]	2.00	0.0380	2.80	0.0068
Virginiamycin s [STR]	1.36	0.2307	3.27	0.0368

a Predicted antibiotic resistance in bile duct aspirates was determined by aligning sequenced reads generated from the phenol chloroform and QIAamp Blood processed samples with (^¶^) and without host DNA depletion to the ResFinder 4.1 database of acquired AMR genes. Detection rates across 22 bile aspirates was determined, and the Wilcoxon test was used to determine whether the use of host DNA depletion had a significant impact on predicting AMR phenotypes. PEN, penicillin; CEPH, cephalosporin; LIN, lincosamide; STR, streptogramin; CARB, carboxypenicillin.

Predicted AMR phenotypes impacted by DNA extraction method and use of host DNA depletion were almost completely independent. The DNA extraction method exclusively influenced detection of aminoglycoside and macrolide resistance, while use of DNA depletion exclusively influenced detection of beta-lactams (penicillins, cephalosporins, carboxypenicillins). Additionally, detection of susceptibility to several lincosamide and streptogramin antibiotics was influenced by both DNA extraction and use of host DNA depletion (Tables 3 and 4).

Resistance to the aminoglycoside and MLS (macrolides, lincosamides, and streptogramins) antibiotics was found only in Gram-positive bacteria (Enterococcus and Streptococcus species). Differential detection of resistance to these antibiotics may be explained by the use of bead beating during DNA extraction. In samples with high abundances of Gram-positive bacteria, resistance was only detected when bead-beating was utilized during DNA extraction, resulting in the protease-lysis-dependent (QIAamp Blood) protocol displaying reduced rates of detection. Use of host DNA depletion with QIAamp Blood processed samples increased the sequence depth of Gram-positive bacteria, and this partially restored the reduced detection rates observed in the QIAamp Blood processed samples by increasing detection of predicted susceptibility to several lincosamide and streptogramin antibiotics.

In contrast, resistance to the beta-lactams was found in Gram-negative bacteria (Klebsiella species, E. coli, E. cloacae, Hafnia alvei), and thus detection of these resistance phenotypes was not influenced by bead-beating. Detection was, however, influenced by host DNA depletion. Host DNA depletion increased detection of beta-lactam resistance in bile samples that had a low relative abundance of microbial reads (<1% of total reads sequenced) and a high dominance of Gram-negative bacteria. Removal of host DNA may have reduced competition for the sequencing pores. This would have increased the number of bacterial reads sequenced, resulting in detection of resistance phenotypes that went undetected when host DNA depletion was not applied.

In the clinical setting, increased detection of penicillin and cephalosporin resistance was of particular interest as SC had detected resistance to these antibiotic classes in 82% (18/22) of bile samples. This indicated that improved detection of resistance to penicillins and cephalosporins may be of clinical value. In contrast, ticarcillin, pristinamycin, quinupristin, and virginamycin are not approved for clinical use by the FDA, and so improved detection of these antibiotics in this setting added no additional value. However, ticarcillin, pristinamycin, and quinupristin are approved for clinical use outside the U.S., suggesting that improved detection of these antibiotics would be of use outside the U.S.

### Detection of chromosomal point mutations conferring antimicrobial resistance.

Chromosomal point mutations conferring AMR were detected in 59% of the bacterial positive samples. The detected point mutations were found in 8 bacterial genes present in the E. coli, Klebsiella pneumoniae, Salmonella enterocolitica, and E. faecium genomes, and resulted in predicted resistance to a number of antibiotic classes, including aminoglycosides (gentamicin, kanamycin, kasugamycin, tobramycin), fluoroquinolones (ciprofloxacin), quinolones (nalidixic acid), and beta-lactams (ampicillin, carbapenem, cephalosporins) (Table 5).

**TABLE 5:** Detection of chromosomal point mutations conferring antimicrobial resistance^*a*^

Gene	Species	Detection rate (*n* samples)	Resistance
*16S rrsB*	Escherichia coli	2	Gentamicin C, Kanamycin A, Tobramycin
*16S rrsC*	Escherichia coli	1	Kasugamycin
*acrR*	Klebsiella pneumoniae	1	Fluoroquinolone
*gyrA*	Salmonella enterica	2	Nalidixic acid, Ciprofloxacin
*ompK36*	Klebsiella pneumoniae	4	Cephalosporins
*ompK37*	Klebsiella pneumoniae	1	Carbapenem
*parC*	Salmonella enterica	1	Nalidixic acid, Ciprofloxacin
*pbp5*	Enterococcus faecium	4	Ampicillin

a Sequenced reads generated from bile aspirates from phenol chloroform processed samples with host DNA depletion were aligned to the Pointfinder (v2.0) database of chromosomal point mutations conferring antimicrobial resistance.

Comparison with SC found no correlation between observed AMR phenotypes and AMR phenotypes predicted as a result of chromosomal point mutations detected in the sequencing data. This was likely due to the limited number of bacterial genomes included in the PointFinder database, with many of the bacterial species detected using culture not being included in the PointFinder database (i.e., E. cloacae, Enterococcus casseliflavus, Finegoldia magna, Streptococcus species, H. alvei, Yersinia enterocolitica, C. freundii, and Proteus hauseri). This indicates that currently PointFinder does not improve AMR detection but has future potential if more bacterial genomes are added.

### Comparison of antimicrobial resistance identification tools.

**FIG 3:**
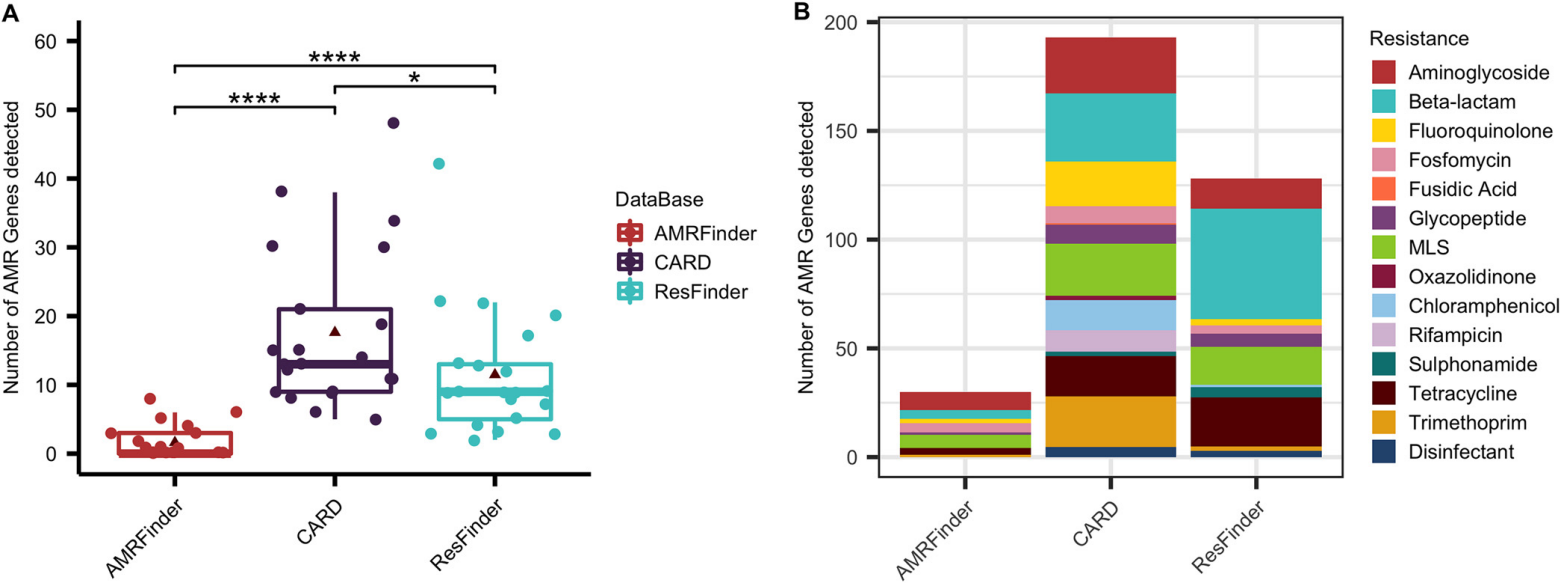
Detection of antimicrobial resistance genes and predicted phenotypes in intraoperative bile duct aspirates using different antimicrobial resistance databases. Sequenced reads generated from the phenol chloroform plus host DNA depletion protocol were aligned to the AMRFinder Plus, CARD, and ResFinder 4.1 databases of antimicrobial resistance (AMR) genes. Total number of AMR genes (A) and predicted AMR phenotypes (B) detected using each database was determined, and statistical analysis was performed using the Wilcoxon test. Solid line, median; triangle, mean; *, *P* value ≤ 0.05; ****, *P* value ≤ 0.00005.

Alignment of the phenol chloroform sequenced reads to different AMR gene reference databases influenced the number of AMR genes detected (*P* value = 6.52e-06), resulting in significantly different predicted AMR profiles (Fig. 3). Alignment to the CARD AMR database resulted in increased detection of AMR genes compared to ResFinder 4.1 (*P* value = 0.0392) and AMRFinder Plus (*P* value = 5e-07), and alignment to the ResFinder 4.1 database resulted in significantly higher number of AMR hits compared to AMRFinder Plus (*P* value = 1.2e-06) (Fig. 3).

In total, 13 antibiotic classes displayed differential AMR prediction rates across the 3 AMR databases. Alignment to the CARD database significantly increased AMR prediction rates compared to AMRFinder Plus and ResFinder 4.1. AMR predictions were increased for 12 antibiotics when CARD was compared to AMRFinder, and 8 AMR predictions were increased when CARD was compared to ResFinder 4.1 (Table 6).

**TABLE 6:** Detection of antibiotic resistance using different databases of antimicrobial resistance genes^*a*^

Antibiotic class	AMRFinder plus *vs* CARD		AMRFinder plus vs ResFinder		CARD vs ResFinder	
	Fold change	*P* value	Fold change	*P* value	Fold change	*P* value
Aminoglycoside	–5.67	<0.0001	–1.67	0.1043	3.40	0.0002
Beta-lactam	–24.00	<0.0001	–27.00	< 0.0001	–1.13	0.9800
Fosfomycin	–4.25	0.0160	0	1.0000	4.25	0.0160
Glycopeptide	–17.00	0.0043	–9.00	0.2896	1.89	0.0693
MLS	–17.80	<0.0001	–8.80	< 0.0001	2.02	0.0180
Chloramphenicol	*–IFN*	<0.0001	*–IFN*	0.1600	26.50	<0.0001
Quinolone	–25.67	<0.0001	–2.00	0.3300	12.83	<0.0001
Sulfonamide	*–IFN*	0.0098	*–IFN*	0.0809	1.17	0.3586
Tetracycline	–18.00	<0.0001	–13.50	< 0.0001	1.33	0.3900
Trimethoprim	–30.67	<0.0001	1.5	0.6500	46.00	<0.0001
Fusidic Acid	*–IFN*	0.1600	-	NA	*IFN*	0.1600
Oxazolidinone	*–IFN*	0.0200	-	NA	*IFN*	0.0200
Rifampin	*–IFN*	<0.0001	-	NA	*IFN*	<0.0001

a Sequenced reads generated from the Phenol Chloroform plus host DNA depletion protocol were aligned to three commonly used AMR gene databases: AMRFinder Plus, CARD, and ResFinder 4.1. The total number of AMR genes detected was determined for each microbial bile aspirate, and statistical analysis was determined using the Wilcoxon test. *IFN*, antibiotic classes where AMR genes were only detected in one group; NA, antibiotic classes where AMR genes were not detected in both groups.

Predicted AMR phenotypes were compared to AMR phenotypes observed using SC to determine the predictive value of NS using each AMR database. Using the AMRFinder Plus database resulted in significantly decreased AMR prediction compared to the CARD and ResFinder 4.1 database (percentage of SC AMR phenotypes predicted = 14.76% versus 78.57% versus 81.19%, *P* values = 4.2e-06 and 1.1e-06, respectively). Comparison between CARD and ResFinder 4.1 found no differences in prediction between the two databases despite alignment to the CARD database resulting in significantly increased detection of AMR genes.

### Time to results.

**FIG 4:**
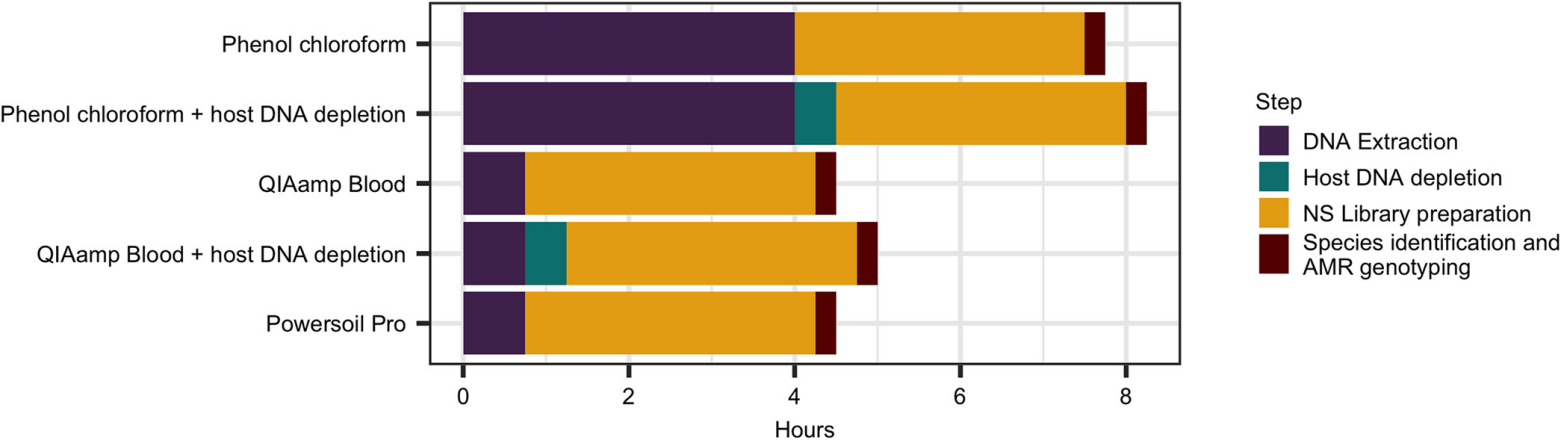
A breakdown of the time taken to detect microbial species and perform AMR genotyping using different methods of DNA extraction and library preparation with nanopore sequencing.

On average it took SC 98 h to complete species identification and AMR typing. In contrast, it took a maximum of 8 h following sample collection to complete DNA extraction and NS library preparation, and species identification with AMR genotyping could be performed within 15 min of initiating the sequence run (Fig. 4). Complete detection of microbial species and AMR genotyping varied depending on sample and NS method used, but on average it took 6 h of sequencing to complete analysis, resulting in an average turnaround time of 14 h. The phenol chloroform method took significantly longer to extract DNA compared to the QIAamp Blood and Powersoil Pro extraction kits (approximately 4 h versus 45 min) (Fig. 4). Use of host DNA depletion took an additional 30 min when preprepared Protein A-bound magnetic beads were used, and library preparation using the Rapid PCR barcoding kit took approximately 3.5 h to complete (Fig. 4).

**FIG 5:**
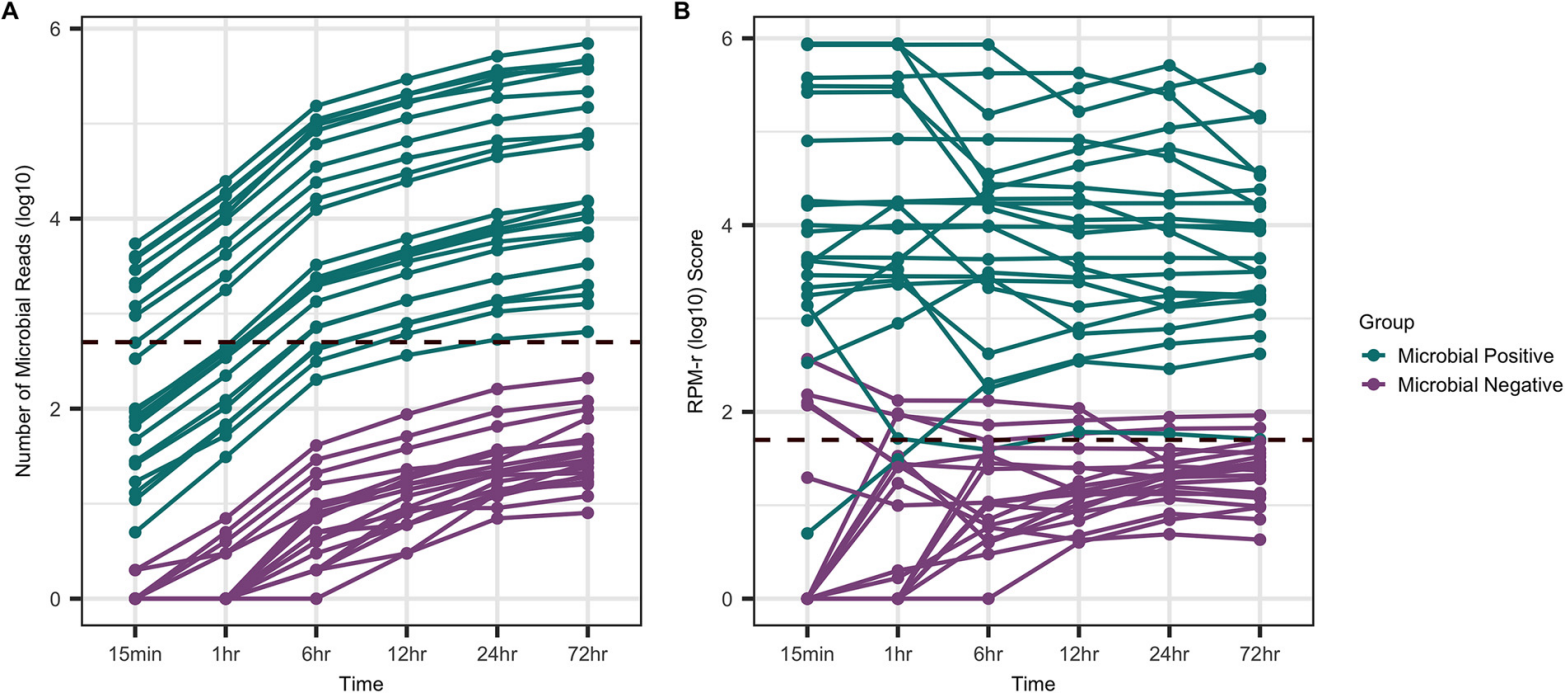
Detection of microbial reads over the course of a 72-h sequence run. To determine the optimum sequencing time required to meet the minimum bacterial read threshold, the number of sequenced bacterial reads was quantified at time points 15 min, 1 h, 6 h, 12 h, 24 h, and 72 h. The quantified reads from both the microbial positive and negative samples were then compared to the minimum bacterial read count threshold (dashed line, A) and minimum RPM-r threshold (dashed line, B) to determine the earliest point in the sequence run that the sample could be declared microbial positive.

NS allows for real-time analysis of sequencing data and the generation of multiple microbial diagnostic reports. A single report containing all diagnostic information, however, is preferable and reduces the likelihood of multiple empirical antimicrobials being administered. The minimum sequence run length required to produce reliable microbial and AMR identification was, therefore, investigated using sequenced data generated from the phenol chloroform methods. Analysis of the number of bacterial reads detected revealed that the microbial positive samples reached the minimum bacterial threshold required to be declared microbial positive at different time points in the sequence run (Fig. 5A). Comparison of microbial positive to microbial negative samples revealed that microbial positive samples generated more microbial reads compared to the microbial negative samples at all stages of the sequence run (Fig. 5A). This suggests that the minimum threshold required to declare a sample microbial positive could be lowered during the early stages of the sequence run (15 min – 6 h) to 250 microbial reads detected (Fig. 5A, log_10_ = 1.35). As for RPM-r scores, most microbial positive samples reached the minimum threshold RPM-r value in the first 15 min of the sequence run (Fig. 5B, RPM-r log_10_ = 1.7). However, at the minimum threshold level, there was some overlap of microbial positive and microbial negative samples, highlighting the need to use multiple parameters when defining the minimum threshold.

Analysis of clinical data from microbial negative samples that had borderline RPM-r scores revealed that four samples came from patients who tested negative for biliary microbes (culture and sequencing negative) but developed an SSI within 30 days postsurgery. This suggests that scoring a borderline RPM-r score may be an early indicator of SSI in intraoperative samples that are culture/sequencing negative.

**FIG 6:**
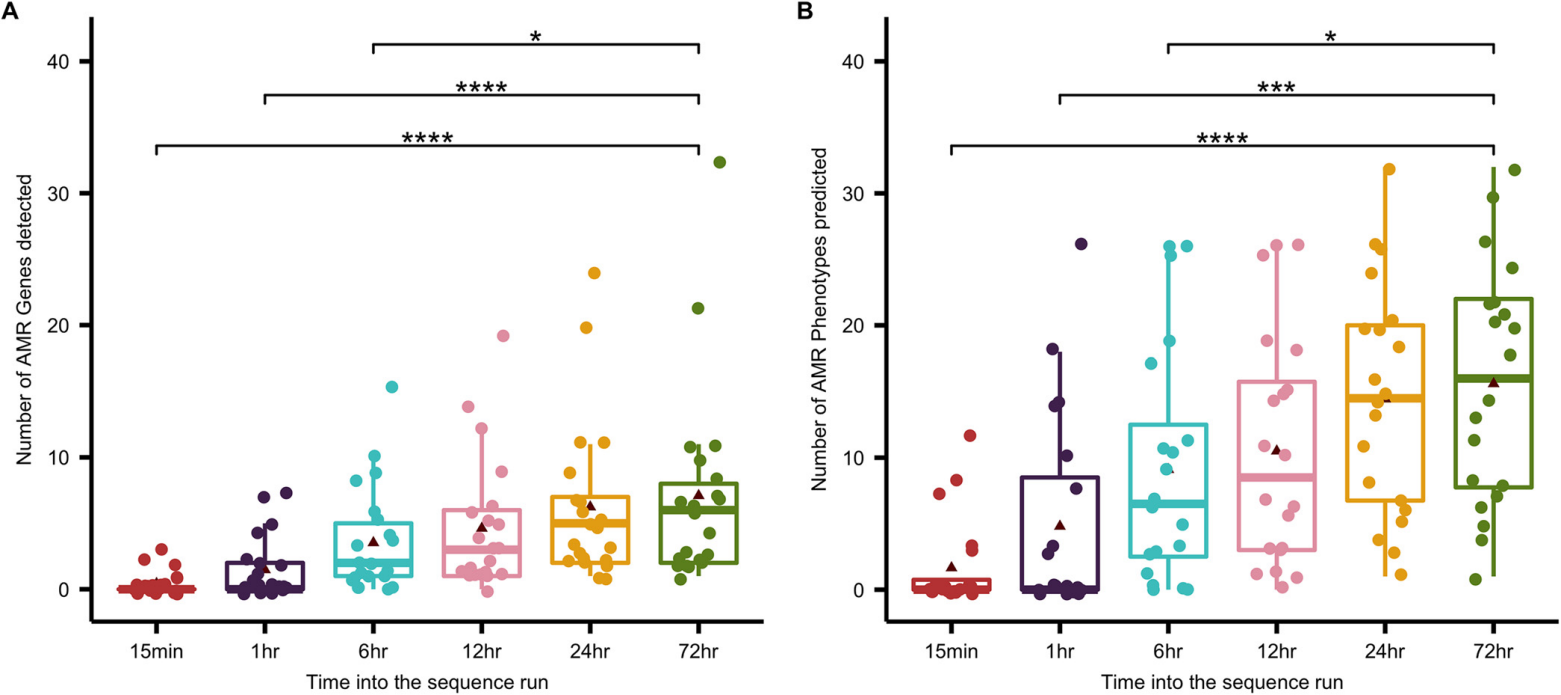
Detection of AMR over the course of a 72-h sequence run. To determine the optimum sequencing time required to detect antimicrobial resistance (AMR) genes and phenotypes, the sequenced reads were concatenated at time points 15 min, 1 h, 6 h, 12 h, 24 h, and 72 h into the sequence run and aligned to the ResFinder 4.1 database of AMR genes and phenotypes. The total number of AMR genes (A) and phenotypes (B) detected in each sample were plotted, and the Kruskal-Wallis H test was used to determine statistical significance in AMR gene and phenotypes detected at the different time points. Solid line, median; triangle, mean; *, *P* value < 0.05; ***, *P* value < 0.0005; ****, *P* value < 0.00005.

Identification of bacterial species was consistent at all time points, indicating that species identification could be called in the first 15 min of the sequence run (Fig. S6). In contrast, the number of detected AMR genes and predicted AMR phenotypes increased over the course of the sequence run (Fig. 6). Time points 15 mins and 1 h were associated with significantly reduced detection of AMR genes and predicted AMR phenotypes, and from time point 12 h there were no significant differences in both the number of AMR genes detected and predicted AMR phenotypes (Fig. 6). Comparison of which AMR phenotypes were predicted at the different time points revealed that most AMR phenotypes were predicted in the first 6 h of the sequence run. When the phenol chloroform method was applied, there was increased prediction of amoxicillin, aztreonam, cefepime, ceftriaxone, and ticarcillin resistance, after the 6-h time point, while use of host DNA depletion resulted in increased prediction of amoxicillin, ampicillin, cephalothin, piperacillin, and ticarcillin resistance after the 6-h time point.

**FIG 7:**
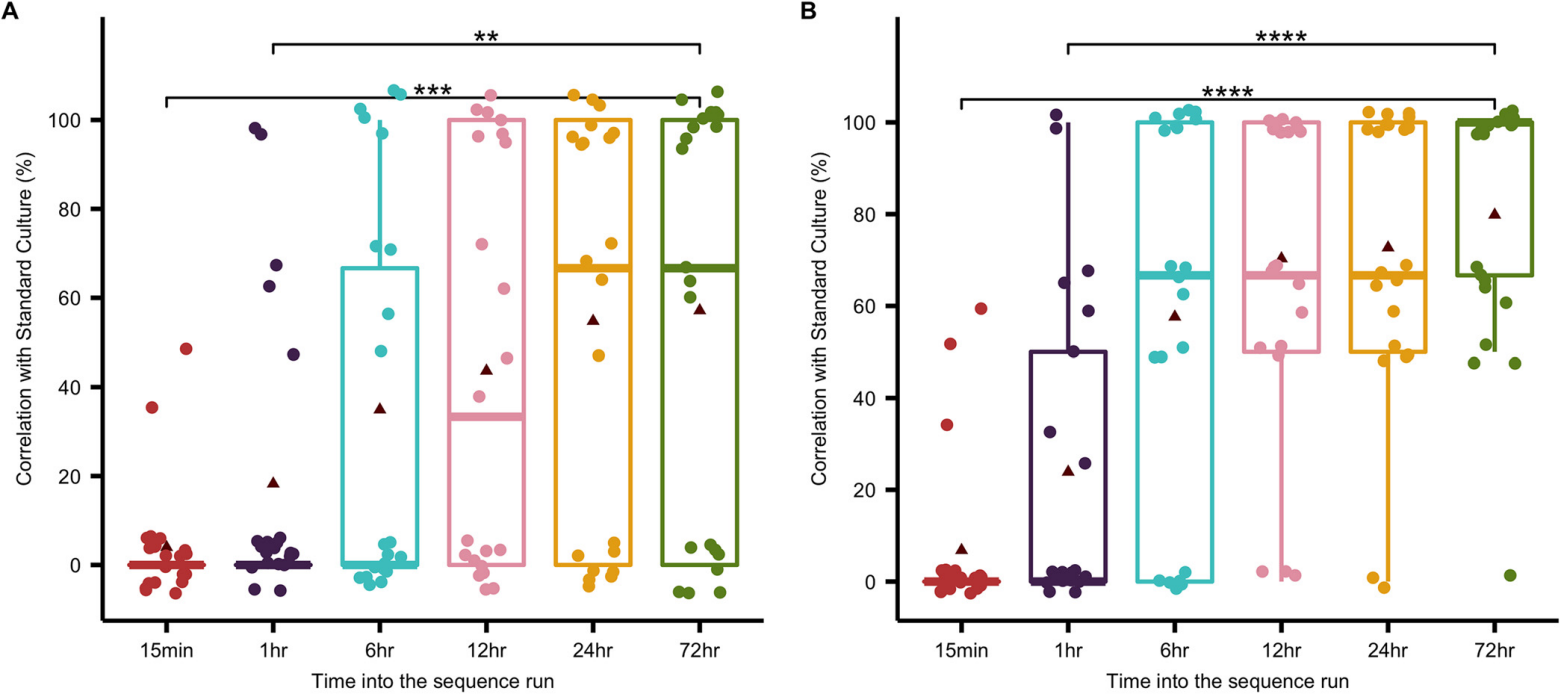
AMR predictive power of nanopore sequencing over the course of a 72-h sequence run. Antimicrobial resistance (AMR) phenotypes were predicted by nanopore sequencing by aligning sequenced reads to the ResFinder 4.1 database of acquired AMR genes. Predictive AMR phenotypes detected using the phenol chloroform method (A) and the phenol chloroform method with host DNA depletion (B) were compared to AMR phenotypes observed using standard culture to determine the predictive power of nanopore sequencing. Data were plotted as percentage of observed AMR phenotypes predicted by nanopore sequencing, and analysis was performed at different time points in the sequence run to determine how predictive power changed over the course of the sequence run. Statistical analysis was performed using the Kruskal-Wallis H test. Solid line, median; triangle, mean; **, *P* value < 0.005; ***, *P* value < 0.0005; ****, *P* value < 0.00005.

Comparison of AMR phenotypes predicted over the course of the sequence run to the resistance phenotypes detected using SC revealed that length of sequence run correlated to NS AMR predictive power (Fig. 7). On average, 4.8% of observed AMR phenotypes were predicted by NS 15 min into the sequence run, 19.8% within 1 h of the sequence run, 36.5% at 6 h into the sequence run, 56.3% at 24 h into the sequence run, and 58.7% at the completion of the sequence run (72 h) (Fig. 7A). Predicted AMR phenotypes were significantly lower at 15 min and 1 h into the sequence run compared to 6 h, 12 h, 24 h, and 72 h, and following 6 h NS AMR predictive power did not significantly improve (Fig. 7). Use of host DNA depletion, while not significantly increasing the average predictive power at any time point into the sequence run, did improve AMR detection across the time points in 62% (13/21) of samples. In 33% (7/21) of samples, use of the phenol chloroform alone failed to detect any observed AMR phenotypes detected using SC. Use of host DNA depletion in these samples increased prediction of observed AMR phenotypes by an average of 57%, with 3 samples having 100% of observed AMR phenotypes detected after host DNA depletion was applied. The samples had the lowest number of microbial reads detected, with an average abundance of 0.61% microbial reads compared to human reads. This indicated that use of host DNA depletion is a critical step in improving AMR detection in samples with a low microbial load.

Overall, these results demonstrate that microbial identification can be determined within the first 15 min of the sequence run, while the majority of the bile resistome can be characterized using a 6-h sequence run. This finding was further supported by SC correlative analysis, whereby the predictive power of NS did not significantly improve after 6 h of sequencing (Fig. 7).

### Surgical site infections.

The SSI incidence rate was 21.4%, with 9 patients developing SSIs within 30 days of pancreaticoduodenectomy or total pancreatectomy surgery. Of these, 44% (4/9) had positive intraoperative bile cultures, and 22% (2/9) had positive intraoperative bile cultures and postoperative abdominal fluid cultures (Table 7). In the patients with positive postoperative cultures, the microbes identified correlated with the species identified from the intraoperative bile, indicating a causal association between biliary microbes and SSI in these patients (Table 7). NS using the phenol chloroform and Powersoil Pro methods identified the postoperative microbial species from the bile aspirates. This suggests that had NS been used to guide antimicrobial tailoring in this study, using NS with either the phenol chloroform method or Powersoil Pro method would have reliably predicted the postoperative bacterial and fungal infections within 24 h postsurgery. This would have been faster than SC, which took 4 days to complete AMR typing of E. faecalis, and 3 and 7 days, respectively, for identifying C. albicans in BA133 and BA141, and may have improved patient outcome.

**TABLE 7:** Microbial species and antimicrobial resistance detected in surgical site infections using standard culture techniques^*a*^

Sample	Intra-operative cultures	Observed AMR phenotypes	Post-operative cultures
BA128	Enterobacter cloacae complex, Citrobacter freundii, Enterococcus faecalis, Klebsiella oxytoca*/* Raoultella ornithinolytica	Ampicillin *(*E. cloacae complex, C. freundii complex), Ampicillin + sulbactam (E. cloacae complex, C. freundii complex), Aztreonam (C. freundii complex), Cefazolin (E. cloacae complex, C. freundii complex), Ceftazidime (C. freundii complex) Ceftriaxone (C. freundii complex)	No growth
BA133	Streptococcus anginosus, Escherichia coli, Proteus hauseri*/ vulgaris, Ligilactoacillus animalis/ murinus, Lacticaseibacillus casei/ paracasei,* Streptococcus constellatus, Enterococcus termitis, Klebsiella oxytoca*/* Raoultella ornithinolytica, Candida albicans	Ampicillin (Proteus hauseri*/ vulgaris,* Klebsiella oxytoca*/* Raoultella ornithinolytica), Cefazolin (*P. hauseri/ vulgaris*), Meropenem (*Lacticaseibacillus casei/ paracasei*)	C. albicans
BA135	Enterococcus faecalis, Klebsiella pneumoniae	Ampicillin (K. pneumoniae), Ampicillin + sulbactam (K. pneumoniae)	No growth
BA141	Bifidobacterium animalis, Streptococcus anginosus, Lactobacillus animalis*/ murinus,* Lactobacillus gasseri, Enterococcus faecium, K. pneumoniae complex, E. faecalis, Hafnia alvei, C. albicans	Ampicillin (K. pneumoniae complex, *H. alvei*), Ampicillin + sulbactam (*H. alvei*), Cefazolin (*H. alvei*)	E. faecalis, C. albicans

a Culture techniques were used to detect intraoperative biliary contamination and antimicrobial resistance in patients undergoing pancreaticoduodenectomy. Postoperative cultures were performed on infectious abdominal fluids from patients who developed surgical site infections.

Additionally, in all four patients, NS predicted significantly higher number of AMR phenotypes compared to the number of AMR phenotypes observed using SC. This has clinical relevance as antibiotic tailoring using SC resulted in patients being prescribed postoperative antibiotics that NS predicted resistance to. This suggests that use of NS AMR prediction in these patients could be a tool to improve antibiotic use by identifying potentially ineffective antibiotics.

## DISCUSSION

Use of prophylactic antibiotics has been demonstrated to reduce rates of SSI, but limitations associated with current techniques used to guide targeted prophylactic therapy has resulted in extensive use of broad-spectrum antibiotics and suboptimal treatment. In this study, we demonstrated that NS can provide a rapid and comprehensive profile of microbial species and associated resistances in intraoperative bile aspirates. NS identified biliary microbes in 100% of samples that were culture positive and detected no microbial species in samples that were culture negative. Additionally, NS detected fungal species in two samples that were negative for fungal growth, indicating increased sensitivity compared to SC. This suggests that the technology could be utilized in the clinical setting to determine which patients should continue with postoperative antimicrobial therapy and which can be potentially taken off treatment. This would reduce the number of surgical patients exposed to broad-spectrum antimicrobial therapy and contribute to antibiotic stewardship. Early de-escalation of postoperative antimicrobial therapy could also reduce the risk of antibiotic-associated adverse events (21, 49, 50) and the health care costs associated with administration of antibiotics (51).

In patients who tested positive for biliary microbes, species identification was achieved within 15 min of sequencing, and AMR genotyping within 6 h, resulting in a time-to-result of approximately 14 h. This was significantly shorter than the average time of 98 h it took to complete microbial identification and AMR typing using SC techniques. However, this analysis was performed in a research capacity and in the clinical setting, in the absence of a pathology lab running 24/7, it is unlikely that the 14-h NS pipeline could be performed immediately after surgery considering that pancreaticoduodenectomy and total pancreatomy are extensive surgeries that on average take 6 h to complete. Obtaining NS results within the first 24 h postsurgery is, therefore, a more likely scenario.

Comparison of NS to SC found that on average NS could identify up to 77% of cultured bacterial species and 76% of cultured fungal species, and predicted up to 81% of observed AMR phenotypes. These results are comparable to previous studies comparing NS and SC results (17, 28, 38, 52), suggesting that NS could be used to guide early antimicrobial therapy following surgery. Given the speed with which NS can identify microbial species and predict AMR phenotypes, future investigations should determine whether NS can be used in replacement of bile cultures for detection and characterization of bile microbes. This would allow targeted antimicrobial therapy to be administered within 24 h postsurgery, reducing the risk of SSI by narrowing the window of opportunity pathogenic species have to grow at the site of surgical incision.

Additionally, early de-escalation of broad-spectrum antimicrobial therapy, either by taking the patient off antimicrobial prophylactic therapy completely or switching to a more targeted approach, may reduce the risk of long-term consequences of broad-spectrum antibiotic use. Use of broad-spectrum antibiotics have been demonstrated to induce gut microbial dysbiosis as a consequence of nonspecific targeting of commensal species, resulting in expansion of pathogenic organisms, such as Clostridium difficile (53, 54). Antimicrobial therapy has also been shown to increase the number of resistance genes harbored in the gut (55–57) and nasal microbiota, subsequently increasing susceptibility to infections due to resistant species (58). Nasal carriage of methicillin-resistant Staphylococcus aureus (MRSA), for example, has been shown to positively correlate to antibiotic usage in Europe (59), and exposure to antibiotics during hospital stay has been associated with increased risk of developing sepsis (60). Use of antibiotics has also been associated with increased risk of cancer (61–64) and heart disease (65–68), although it should be noted that these data are correlative rather than causative.

NS enables simultaneous detection of all known AMR genes in a single test. Increased depth of AMR typing has the potential to improve antimicrobial therapy by enabling a more targeted approach. In a recent 10-week clinical trial, same-day NS results were used to make changes to antibiotic treatment of bacterial respiratory infection in a UK intensive care unit (17). In the majority of cases, NS results resulted in one of more antibiotics being stopped completely or a more narrow-spectrum treatment being administered (17). Given that 30–90% of antibiotics prescribed for surgical prophylaxis have been reported as inappropriate, used at the wrong time, or administered for excessively long periods and with a too broad-spectrum coverage (69), use of NS to improve current antimicrobial regimens is of significant interest. Reducing use of broad-spectrum therapies would contribute toward reducing the spread of AMR. With resistance to almost all antibiotics being reported (70) and an estimated 70% of human pathogens predicted to harbor at least one AMR gene (71, 72), development of tools and techniques to reduce the spread of AMR is essential.

Our study demonstrated that the method of DNA extraction, use of host DNA depletion, and the specific AMR gene database used, significantly influenced microbial identification and prediction of AMR. Use of mechanical lysis using bead beating improved bacterial and fungal species identification. This finding has been observed in previous studies (73–75), and it has been shown that differences in bacterial cell walls are responsible for varying degrees of lysis efficiency (76, 77). In general, it is expected that some level of bias will be observed, whereby Gram-positive bacteria are underrepresented and Gram-negative bacteria are overrepresented in polymicrobial samples, as a consequence of the thicker Gram-positive cell wall (76–78). Reduced detection of Gram-positive bacteria is clinically significant as some of the most common multiresistant bacterial species in the U.S. are Gram-positive, including MRSA, vancomycin-resistant enterococci (VRE), and Clostridium difficile (79). Detection of Gram-positive bacteria and their resistance phenotypes is therefore a crucial component in microbial diagnostics. Improved fungal detection using bead-beating was particularly relevant to the patient group under investigation as the global rate of fungal biliary contamination in patients undergoing pancreaticoduodenectomy has been estimated at 25% (80).

The QIAamp Blood method was also associated with reduced detection of aminoglycoside and MLS resistance genes, while use of host DNA depletion increased detection of AMR genes conferring resistance to beta-lactams and tetracyclines. Reduced detection of AMR genes was likely due to decreased extraction of Gram-positive DNA in samples processed by the QIAamp Blood method. Use of AMR identification tools significantly influenced the number of AMR genes detected, but this did not influence the ability of NS to predict AMR phenotypes detected using SC techniques.

Another key difference in the extraction methods was that the phenol chloroform method was performed on a cellular pellet whereas the two extraction kits were performed on bile aspirate. DNA extracted using the phenol chloroform method was therefore almost exclusively cellular, while DNA extracted using the two extraction kits was a mixture of cellular and cell-free DNA. Bile can induce DNA degradation (81, 82), and thus cell-free DNA extracted from the bile using the extraction kits may have been degraded due to exposure to bile acids, potentially contributing toward differences in species identification across the different DNA extraction methods. Additionally, bead beating has been demonstrated to induce DNA fragmentation, potentially influencing species identification and prediction of AMR. Further investigation into the effects of DNA fragmentation/degradation on species identification and AMR gene detection will be essential in determining the optimum method of DNA preparation for quality NS.

Use of mechanical lysis using bead beating was also found to increase detection of AMR genes conferring resistance to 16 antibiotics. Similarly, use of host DNA depletion resulted in increased prediction of 17 resistant phenotypes, but this only improved NS AMR predictive power for the QIAamp Blood samples. The polymicrobial nature of microbes detected in the bile, variation between samples, and the relatively small study size likely contribute toward the lack of statistical significance. In samples dominated by Gram-positive bacteria, for example, use of beat beating did improve correlation with AMR phenotypes observed by SC, while use of DNA depletion improved correlation to AMR phenotypes observed by SC in samples with low bacterial loads. Use of bead beating with host DNA depletion was, therefore, the most advantageous method of performing unbiased AMR genotyping using AMR technology.

AMR predictions were also found to be influenced by use of AMR gene database. The total number of AMR genes detected was highly variable according to the AMR database used, resulting in significantly altered prediction of AMR. Differences in AMR prediction identified by CARD and ResFinder were due to differences in database construction. ResFinder 4.1 is a database of acquired AMR genes (83), while CARD includes resistant mutations in housekeeping genes and efflux overexpression in addition to acquired AMR genes (84). Positive predictive value of both databases, however, was identical, and predicted AMR by both databases was comparable to SC, predicting 79% and 81% of observed AMR phenotypes, respectively. This suggests that either database may be used to predict AMR resistance and that decisions on which database would be determined by other factors, such as speed of the analysis.

The use of NS in microbial diagnostics is a recent field of enquiry, with the majority of published research proof-of-concept studies that have been published in the last 4 years. There are multiple options for DNA extraction, host DNA depletion, and sequence library preparation, and this has resulted in a lack of standardization with regard to the NS pipeline (85). As evidenced by this study, protocol design may induce bias with regard to species identification and AMR typing. Moreover, the Oxford Nanopore commercial barcoding primers, which are frequently used in NS of low biomass samples, have been demonstrated to reduce detection capacity for some bacterial taxa in complex clinical samples. The 16S primers used in the Oxford Nanopore 16S amplification kits, for example, have been shown to exhibit mismatches resulting in decreased detection of Pseudomonas (86) and Corynebacterium (87, 88). This highlights that while use of NS in microbial diagnostics is promising, development of standardized robust protocols is key if the technology is to be used in the clinical setting.

In summary, this study demonstrated that NS not only rapidly detects microbial species and AMR, but that with the right protocol selection, yields results comparably to SC. These findings suggest that NS could be used to guide early targeted antimicrobial therapy and has the potential to significantly reduce rates of SSI. Moreover, if NS is proven to be a safe and valid diagnostic tool, it could improve antibiotic stewardship by significantly decreasing the duration of broad-spectrum, nontargeted antimicrobial therapy that surgical patients are currently exposed to. This would limit the risks associated with antibiotic use, both to the patient and to the wider community.

## MATERIALS AND METHODS

### Bile collection.

Bile was collected from a previously described cohort of 42 patients who underwent pancreaticoduodenectomy or total pancreatectomy at a single institution (48). In brief, a bile duct aspirate was collected just prior to bile duct division using a 16-gauge needle and a 3-cc syringe, and bile duct swabs were collected after duct division using a Becton, Dickinson BBL CultureSwab inserted into the cut end of the bile duct. Bile aspirate and swab were transported to the clinical microbiology laboratory for SC and AMR typing, and NS was performed on a second bile aspirate sample. Standard of care laboratory procedures (SC, AMR typing) and NS analysis were performed simultaneously, and standard of care results were used to guide antimicrobial therapy postsurgery.

### DNA extraction.

DNA was extracted from bile aspirate within 24 h of sample collection using three forms of DNA extraction: phenol chloroform extraction, bead beating cell lysis with silica membrane DNA extraction, and protease cell lysis with silica membrane DNA extraction. Phenol chloroform extraction was performed using an optimized protocol (Text S1), and a negative control, whereby bile aspirate was replaced with PBS, was generated for each DNA extraction.

DNA extraction using a filter membrane was achieved using two Qiagen DNA extraction kits: the QIAamp Blood DNA minikit (QIAamp Blood) (Qiagen, cat. 51104), which utilizes an initial protease cell lysis step prior to silica membrane DNA extraction, and the Qiagen DNeasy Powersoil Pro kit (Powersoil Pro) (Qiagen, cat. 47016), which utilizes an initial bead beating cell lysis prior to silica membrane extraction. Both kits were performed using recommended protocols, and negative controls were generated for each extraction using kit elution buffer in replacement of bile aspirate. Statistical analysis was performed using Wilcoxon test to determine how effective the three DNA extraction approaches were, and to assess the impact of host DNA depletion of DNA concentration.

### Library preparation and sequencing.

The NEBNext Microbiome DNA Enrichment kit (NEB, cat. E2612) was used to deplete host DNA from the metagenomic DNA. The phenol chloroform method extracted sufficient DNA from 39/42 samples to enable host DNA depletion to be performed, while the QIAamp Blood kit extracted sufficient DNA to enable host DNA depletion to be performed on 33/42 samples. The Powersoil Pro kit failed to generate sufficient DNA to enable host DNA depletion to be performed.

AMPure XP beads (Agencourt, cat. A63881) at a ratio of 1.8× beads to sample were used to purify host depleted genomic DNA. Negative controls were generated using nuclease-free water in replacement of genomic DNA. DNA quantification was performed using a Qubit 2.0 fluorometer (Thermo Fisher Scientific) and the Qubit dsDNA high sensitivity kit (Thermo Fisher Scientific, cat. Q32851). Library preparation was performed using the Oxford Nanopore Technologies Rapid PCR Barcoding Sequencing kit (ONT, cat. SQK-RPB004) according to the manufacturer’s recommended protocol, and sequencing was performed in batches of 5 using R9 flow cells.

### Microbial identification using nanopore sequencing.

Fastq files generated from the basecalled reads were concatenated, and Porechop (version 0.2.3) was used to remove adaptor sequences. Low complexity reads were removed using BBMap (version 38.73, bbduk.sh). This involved filtering out reads having a length less than160 bp, an entropy less than 0.855, or a qin score less than 33. Microbial identification was performed with Centrifuge software (1.0.4) using a customized genome database that contained the human genome along with bacterial, fungal, and viral genomes of known human commensal and pathogenic organisms. Taxa were determined using an in-house script, and bile aspirates were declared microbial positive when 500 or more microbial reads were detected. Additionally, 50 reads per million (RPM) ratio (RPM-r), defined as RPM-r = RPM_sample_/RPM_negative control_, was used as an additional minimum threshold to reduce biases caused by different sequence depth and mitigate concerns regarding potential microbial contamination, as described in previous studies (28, 41, 89, 90).

Relative abundance of detected microbial taxa was determined and statistical analysis using the Wilcoxon test was performed to determine the impact of the different DNA extraction and preparation approaches on the level of microbial detection. For bacterial analysis, this involved assessing the percentage of sequenced reads that aligned to bacterial taxa, while fungal analysis was performed on total number of fungal reads.

### Identification of antimicrobial resistance using nanopore sequencing.

Prediction of AMR phenotypes was performed using AMRFinder Plus, the Comprehensive Antibiotic Resistance Database (CARD), and Resfinder 4.1. ResFinder 4.1 utilized a database of acquired AMR genes to predict AMR phenotypes (83), AMRFinder Plus used a database of antimicrobial resistance genes and some AMR-conferring point mutations (91), and the database used by CARD covered a variety of AMR determinants including acquired AMR genes, resistance associated mutations of housekeeping genes, and efflux pump genes (84, 92).

AMRFinder Plus 3.10.18 was run using the “--plus” option to enable detection of virulence genes and stress tolerance genes in addition to AMR genes (91). AMR hits were defined as reads that aligned to AMR genes with a minimum coverage of 60% and minimum sequence identity of 80%. CARD was run using the default settings and CARD’s Resistance Gene Identifier (RGI) 4.2.2 with the CARD 3.0.1 database. Only “Perfect” and “Strict” AMR hits were included as determined by curated similarity cut-offs, whereby “perfect” AMR hits were an exact match to curated reference sequences and “strict” hits are previously unknown variants of known AMR gene sequences (84, 92). ResFinder 4.1 was run using the ResFinder database of acquired AMR genes with a minimum coverage of 60% and a minimum sequence identity of 80% (83). PointFinder was also run using the PointFinder (v2.0) database and the Campylobacter, Enterococcus faecium, Escherichia coli, Klebsiella, Salmonella, and Staphylococcus aureus schemes, using a minimum coverage of 60% and a minimum sequence identity of 80% (83). AMR analysis was performed on DNA sequences and thus AMR gene expression was not assessed. Detection of AMR genes, therefore, was used to infer likely AMR phenotypes present in the bile microbial populations and are henceforth referred to as predicted AMR phenotypes.

### Time to results.

To determine the optimum time point during the sequence run at which reasonable deductions regarding detection of potential pathogens can be made, python (version 3.6.9, timefilt.py) was used to generate concatenated fastq files containing reads produced during the first 15 min of the sequence run, the first hour, the first 6 h, the first 12 h, the first 24 h, and the full sequence run (72 h). The time point fastq files were analyzed for the presence of microbial DNA and acquired AMR genes using the ResFinder 4.1 database.

### Correlation to standard culture.

NS detection of biliary contamination, microbial species, and AMR was compared to SC results to determine the positive predictive value of the different NS protocols utilized. This was defined as the percentage of cultured microbial species and observed AMR phenotypes detected using NS technology. Patient outcome was collected from chart review, and NS results were compared to postoperative SC results to determine whether NS could predict causative pathogen in patients who developed SSIs.

### Data availability.

Nanopore read data generated from bile aspirates and kit negative controls are available at the Sequence Read Archive under accession no. PRJNA799127.

10.1128/msphere.00964-21.1 TEXT S1 DNA extraction using the phenol chloroform method. Download Text S1, DOCX file, 0.01 MB. Copyright © 2022 Whittle et al. 2022 Whittle et al. https://creativecommons.org/licenses/by/4.0/ This content is distributed under the terms of the Creative Commons Attribution 4.0 International license.

10.1128/msphere.00964-21.2 FIG S1 Comparison of quantity of DNA extracted from intraoperative bile aspirates using different DNA extraction and preparation methods. Genomic DNA was extracted from intraoperative bile aspirates using a phenol chloroform method and the QIAamp Blood and Powersoil Pro kits. Host DNA was removed, and DNA concentration was determined using Qubit 2.0 and the dsDNA HS kit. Statistical analysis was performed using the Wilcoxon test. Solid line, median; triangle, mean; *, *P* value < 0.05; ****, *P* value < 0.00005. Download FIG S1, TIF file, 0.5 MB. Copyright © 2022 Whittle et al. 2022 Whittle et al. https://creativecommons.org/licenses/by/4.0/ This content is distributed under the terms of the Creative Commons Attribution 4.0 International license.

10.1128/msphere.00964-21.3 FIG S2 Number of microbial reads detected in the negative controls. Negative controls were generated for each DNA extraction and host DNA depletion performed. Once generated, the negative controls underwent all the downstream processes as the bile DNA samples. The extracted DNA was sequenced using NS and aligned to microbial genomes to identify potential microbial contamination. Download FIG S2, TIF file, 0.4 MB. Copyright © 2022 Whittle et al. 2022 Whittle et al. https://creativecommons.org/licenses/by/4.0/ This content is distributed under the terms of the Creative Commons Attribution 4.0 International license.

10.1128/msphere.00964-21.4 FIG S3 Principal component analysis of microbial populations detected in intraoperative bile aspirates using different methods of DNA extraction and preparation. Principal component analysis (PCA) was performed at the bacterial species level from 22 intraoperative bile aspirates that tested positive for bacterial contamination. Bacterial populations detected using the phenol chloroform method (with and without host DNA depletion), the QIAamp Blood method (with and without host DNA depletion), and the Powersoil Pro method were normalized using relative abundance scores and plotted. The PCA was performed using Euclidean distance and R software. Each data point represents the bacterial population profile of a bile aspirate, and the distance between two plotted points is proportional to the degree of similarity between the two bacterial populations. Download FIG S3, TIF file, 0.9 MB. Copyright © 2022 Whittle et al. 2022 Whittle et al. https://creativecommons.org/licenses/by/4.0/ This content is distributed under the terms of the Creative Commons Attribution 4.0 International license.

10.1128/msphere.00964-21.5 FIG S4 Principal component analysis of microbial populations detected in the negative controls compared to the bile aspirate samples. Principal component analysis (PCA) was performed at the bacterial species level from 22 intraoperative bile aspirates that tested positive for bacterial contamination, and their corresponding negative controls. Bacterial populations detected using the phenol chloroform method (with and without host DNA depletion), the QIAamp Blood method (with and without host DNA depletion), the Powersoil Pro method, and the NEBNext Microbiome enrichment kit were normalized using relative abundance scores and plotted. The PCA was performed using Euclidean distance and R software. Each data point represents the bacterial population profile of a bile aspirate/negative control, and the distance between two plotted points is proportional to the degree of similarity between the two bacterial populations. Download FIG S4, TIF file, 0.9 MB. Copyright © 2022 Whittle et al. 2022 Whittle et al. https://creativecommons.org/licenses/by/4.0/ This content is distributed under the terms of the Creative Commons Attribution 4.0 International license.

10.1128/msphere.00964-21.6 FIG S5 Relative abundance of bacterial species and human DNA detected in the negative controls. DNA detected in the DNA extraction (Phenol Chloroform, QIAamp Blood, Powersoil Pro) and host DNA depletion (NEBNext enrichment) negative controls were aligned to a database of microbial genomes and the human genome in order to determine the origins of any contaminating DNA. Download FIG S5, JPG file, 1.3 MB. Copyright © 2022 Whittle et al. 2022 Whittle et al. https://creativecommons.org/licenses/by/4.0/ This content is distributed under the terms of the Creative Commons Attribution 4.0 International license.

10.1128/msphere.00964-21.7 FIG S6 Average relative abundance of bacterial species detected at different time points in one 72-hr nanopore sequence run. To determine whether detection of bacterial species alters over the course of a 72-hr sequence run, bacterial species analysis was performed 15 minutes into a nanopore sequence run, 1 hr into the sequence run, 6 hr, 12 hr, 24 hr, and 72 hr. Download FIG S6, TIF file, 0.9 MB. Copyright © 2022 Whittle et al. 2022 Whittle et al. https://creativecommons.org/licenses/by/4.0/ This content is distributed under the terms of the Creative Commons Attribution 4.0 International license.

10.1128/msphere.00964-21.8 TABLE S1 Average relative abundance of bacterial genera and species detected in bile aspirate using different methods of DNA extraction and use of host DNA depletion. Sequenced genomic reads generated using nanopore sequencing with the phenol chloroform method (with and without use of host DNA depletion), the QIAamp Blood method (with and without use of host DNA depletion), and the Powersoil Pro method, were aligned to a database containing the human genome and genomes of common human pathogens and commensal bacterial species to determine average relative abundance of bacterial species detected using the different methods. Statistical analysis was performed using one-way ANOVA. Download Table S1, DOCX file, 0.02 MB. Copyright © 2022 Whittle et al. 2022 Whittle et al. https://creativecommons.org/licenses/by/4.0/ This content is distributed under the terms of the Creative Commons Attribution 4.0 International license.
