# Supplementary material for: Optimizing Nanopore Sequencing for Rapid Detection of Microbial Species and Antimicrobial Resistance in Patients at Risk of Surgical Site Infections
Source: mSphere. 2022 Feb 16;7(1):e00964-21. doi: 10.1128/msphere.00964-21 (PMC8849348; doi:10.1128/msphere.00964-21)
Supplement: TEXT S1 [file msphere.00964-21-s0001.docx]

**DNA extraction using the phenol chloroform method**

Phenol chloroform extraction was performed using an optimized protocol and involved the following steps. 1ml of bile aspirate was centrifuged for 30 minutes to generate a cell pellet. The bile supernatant was removed, and the cell pellet was resuspended in 500μl buffer A (5M NaCl, 1M Tris, 0.5M EDTA), 500μl phenol-chloroform, and 210μl of SDS. Cell lysis was then performed using two cycles of bead beating (60 seconds at a speed of 60m/sec) with a FastPrep 24 5G machine. The aqueous phase containing the genomic DNA was separated from the cellular proteins, lipids, and other debris using a 3 minute centrifugation step. The aqueous phase was mixed with 500μl of phenol-chloroform and a 3 minute centrifugation step was used to separate the aqueous and organic phases. DNA precipitation was then carried out by incubating the aqueous DNA solution with 600μl of isopropanol on ice for 1 hour. A DNA pellet was then generated using a 20 minute centrifugation step, and the DNA pellet was washed using 500μl of ethanol. Precipitated DNA was resuspended in 200μl of PBS and purified using the QIAamp DNA mini kit (QIAGEN). All centrifugation steps were performed at 4^o^C and maximum speed (14,000 RPM), and a negative control, whereby bile aspirate was replaced with PBS, was generated for each DNA extraction.
