# Supplementary figures and images for: Optimizing Nanopore Sequencing for Rapid Detection of Microbial Species and Antimicrobial Resistance in Patients at Risk of Surgical Site Infections
Source: mSphere. 2022 Feb 16;7(1):e00964-21. doi: 10.1128/msphere.00964-21 (PMC8849348; doi:10.1128/msphere.00964-21)

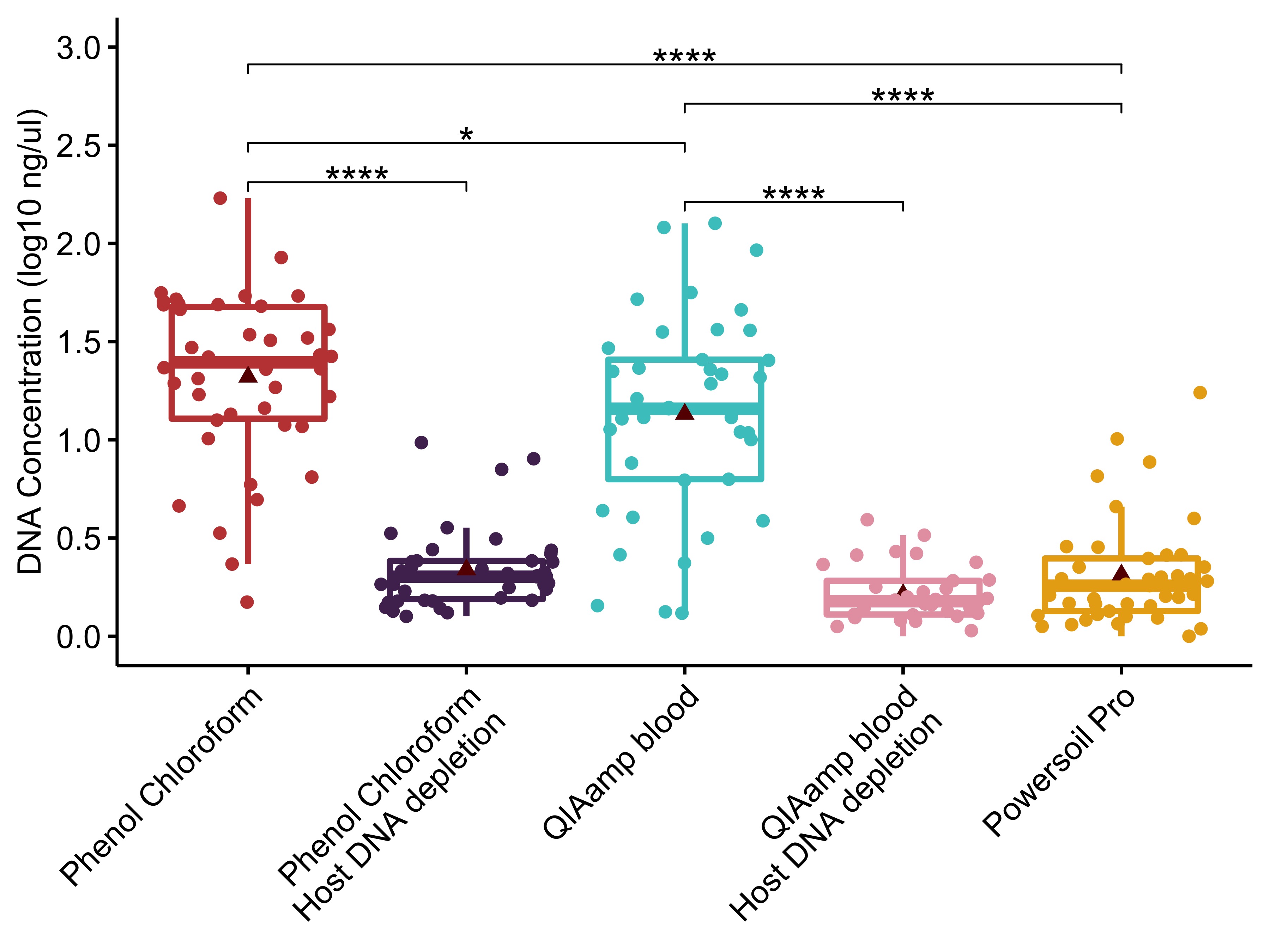

Supplement: FIG S1 [file msphere.00964-21-sf001.tif]

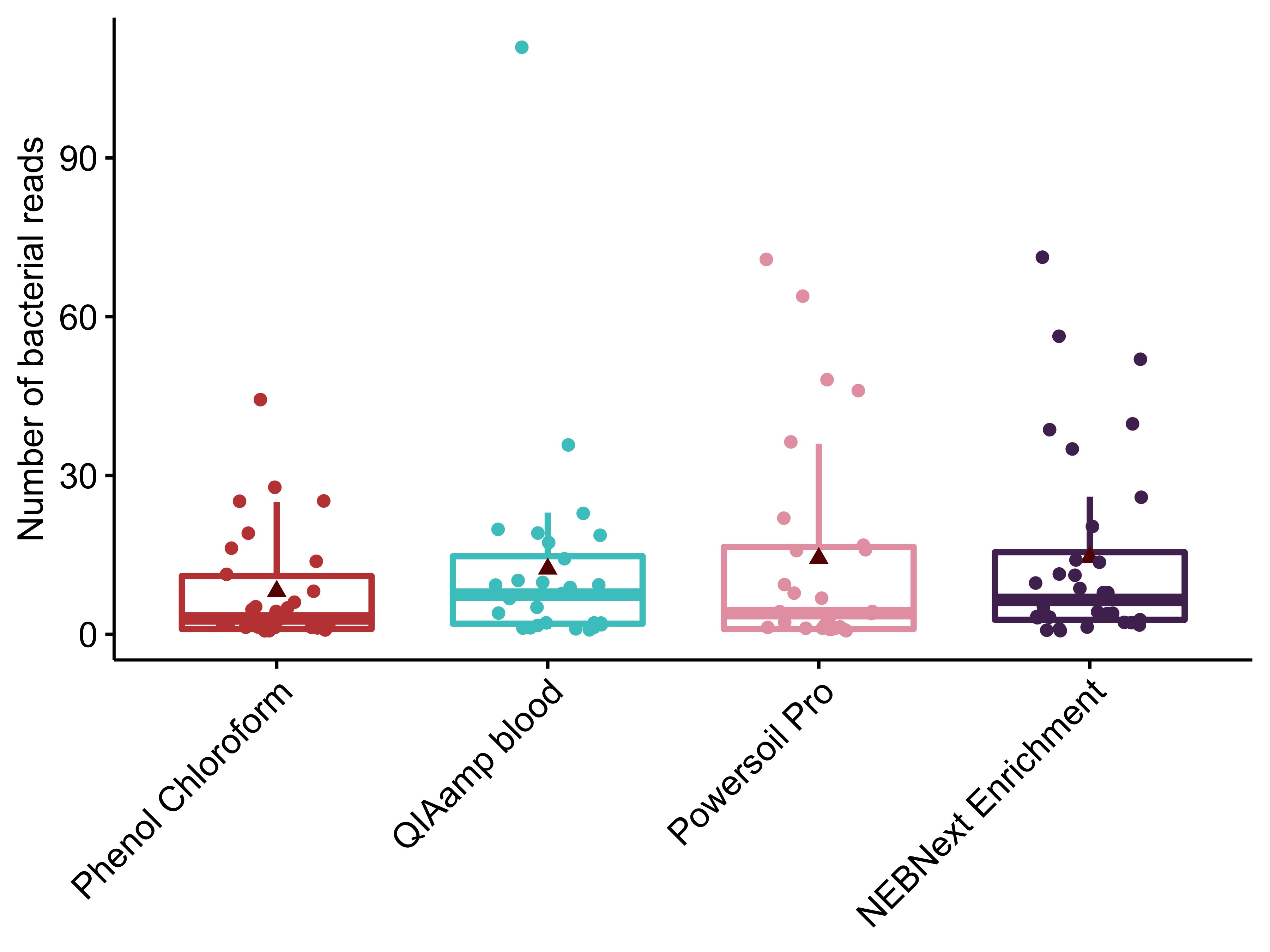

Supplement: FIG S2 [file msphere.00964-21-sf002.tif]

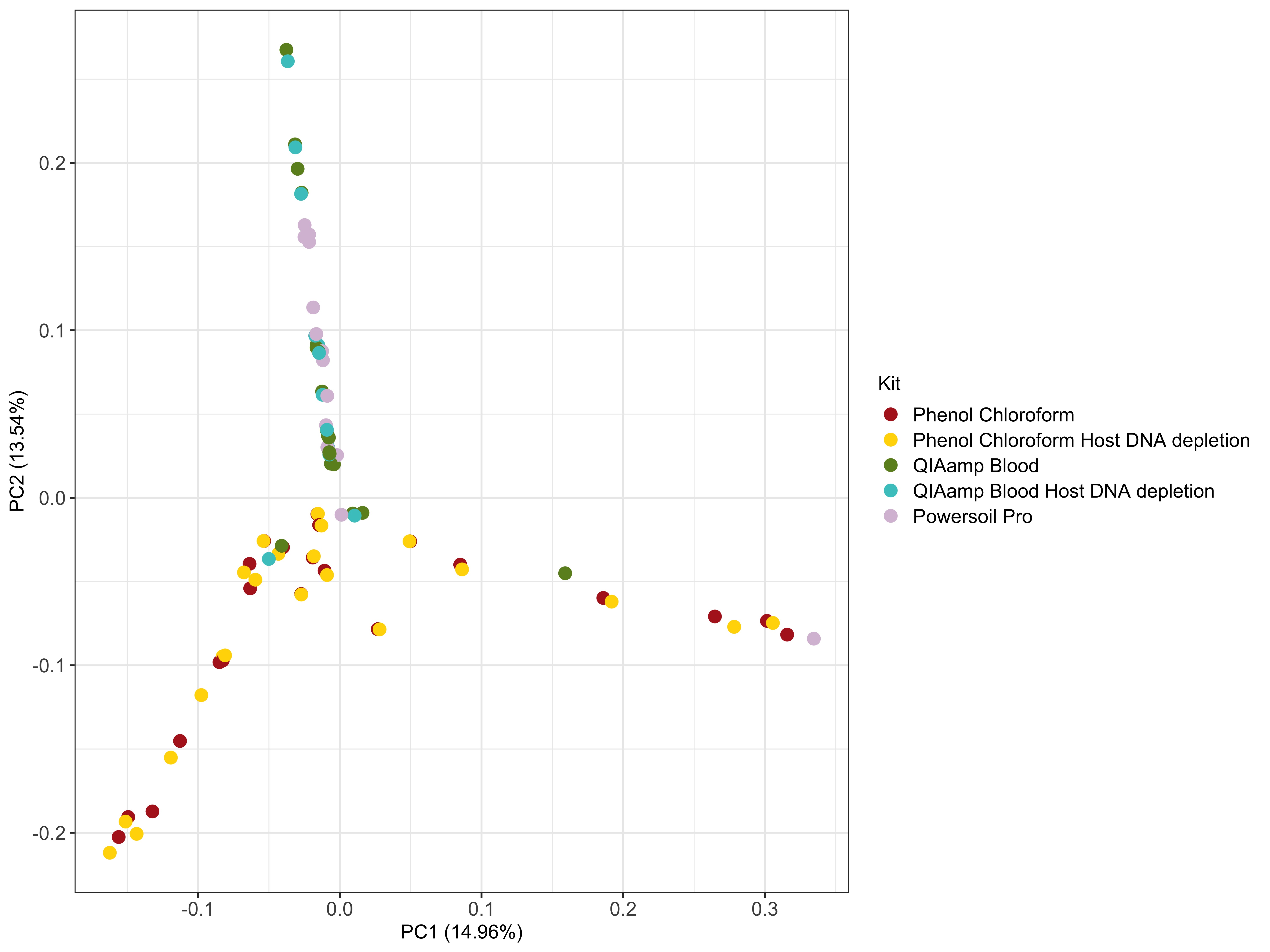

Supplement: FIG S3 [file msphere.00964-21-sf003.tif]

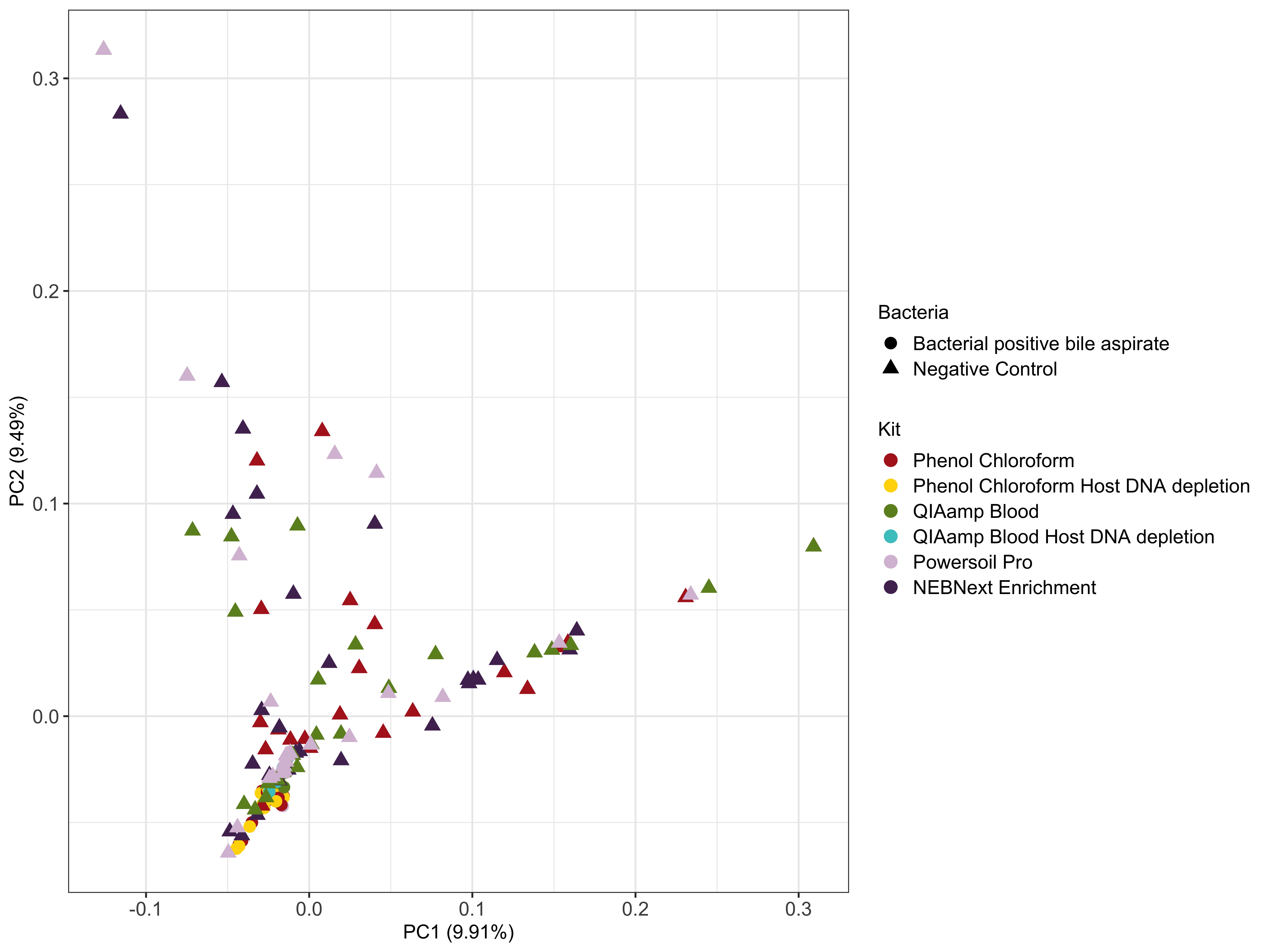

Supplement: FIG S4 [file msphere.00964-21-sf004.tif]

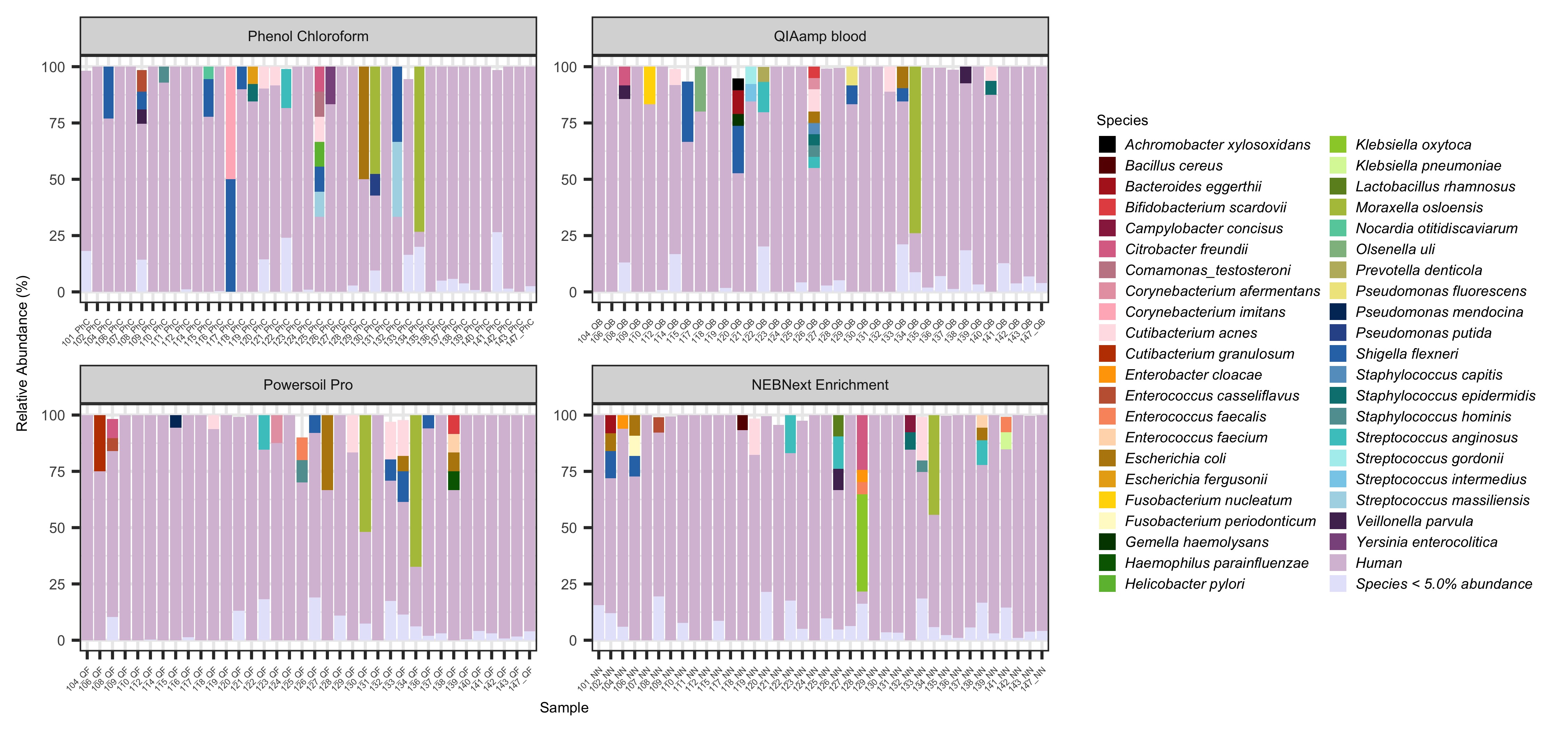

Supplement: FIG S5 [file msphere.00964-21-sf005.jpg]

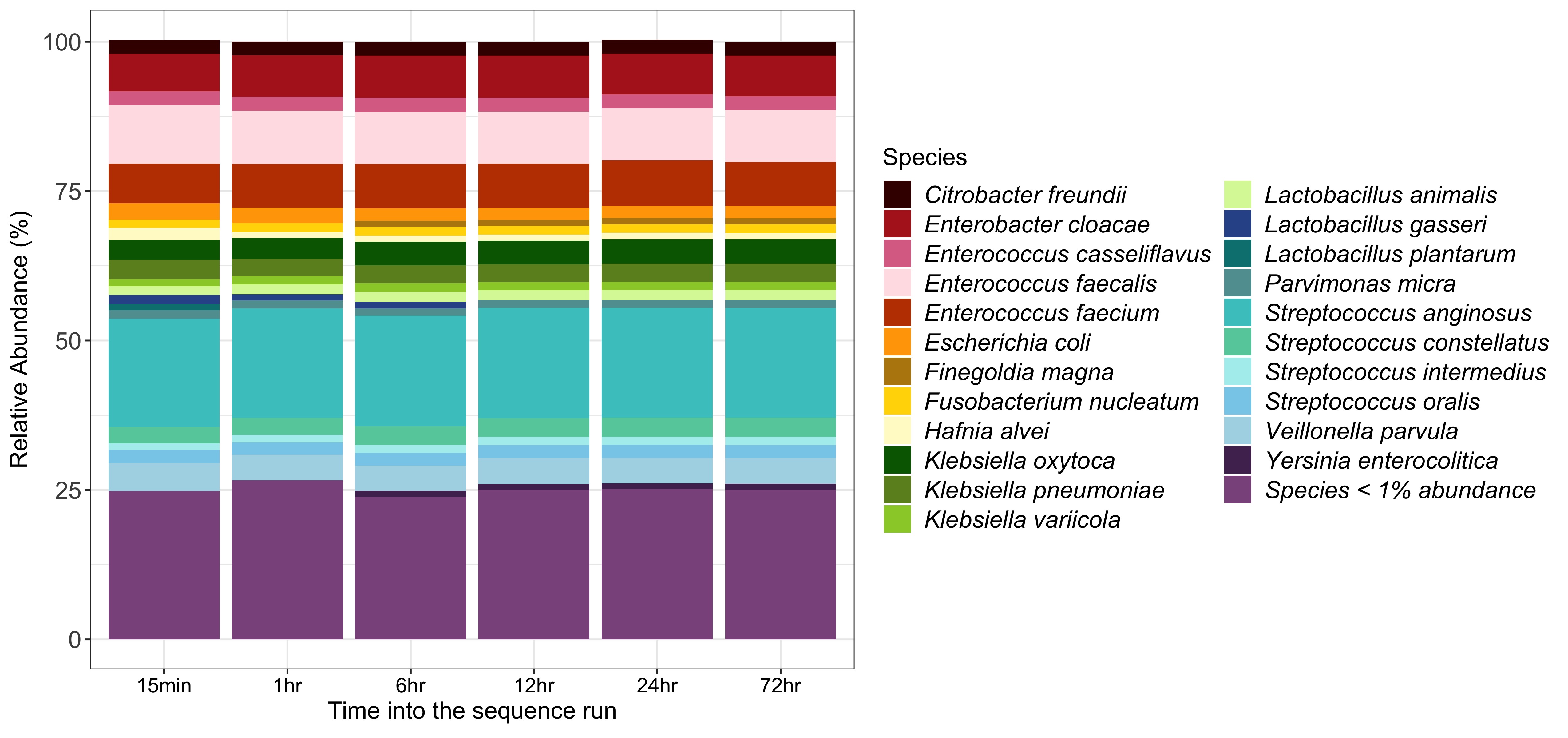

Supplement: FIG S6 [file msphere.00964-21-sf006.tif]
