# Supplementary material for: Optimizing Nanopore Sequencing for Rapid Detection of Microbial Species and Antimicrobial Resistance in Patients at Risk of Surgical Site Infections
Source: mSphere. 2022 Feb 16;7(1):e00964-21. doi: 10.1128/msphere.00964-21 (PMC8849348; doi:10.1128/msphere.00964-21)
Supplement: TABLE S1 [file msphere.00964-21-st001.docx]

| Species | Average percentage of bacterial reads detected (standard deviation) | | | | |  |
| --- | --- | --- | --- | --- | --- | --- |
|  | Phenol chloroform | Phenol chloroform + host DNA depletion | QIAamp Blood | QIAamp Blood +  host DNA depletion | Powersoil Pro | *P value* |
| *Aggregatibacter* | 0.61 (2.08) | 0.58 (1.98) | 1.05 (2.06) | 1.94 (4.83) | 0.74 (1.74) | 0.577 |
| *A. aphrophilus* | 0.52(1.93) | 0.50 (1.86) | 0.74 (1.97) | 1.57 (4.59) | 0.59 (1.65) | 0.740 |
| *Bifidobacterium* | 3.25 (5.94) | 3.48 (6.30) | 0.20 (0.30) | 0.26 (0.30) | 4.46 (6.75) | 0.049 |
| *B. dentium* | 0.82 (1.19) | 0.87 (1.29) | 0.08 (0.16) | 0.14 (0.20) | 1.02 (1.33) | 0.016 |
| *Campylobacter* | 0.88 (3.08) | 0.72 (2.30) | 1.23 (2.77) | 1.80 (3.76) | 1.04 (3.39) | 0.875 |
| *C. showae* | 0.69 (2.80) | 0.54 (2.12) | 0.87 (2.69) | 1.24 (3.45) | 0.83 (3.14) | 0.967 |
| *Citrobacter* | 2.58 (5.08) | 2.40 (4.81) | 3.37 (6.48) | 6.16 (10.40) | 3.10 (5.54) | 0.498 |
| *C. freundii* | 2.40 (4.89) | 2.24 (4.73) | 2.97 (6.34) | 5.75 (10.33) | 2.92 (5.45) | 0.555 |
| *Enterobacter* | 6.92 (18.83) | 6.82 (18.32) | 8.57 (20.09) | 3.17 (5.58) | 8.95 (22.56) | 0.914 |
| *E. cloacae* | 6.92 (18.42) | 6.82 (18.32) | 7.49 (20.08) | 3.17 (5.58) | 8.95 (22.56) | 0.914 |
| *Enterococcus* | 20.39 (24.64) | 21.08 (24.80) | 8.49 (18.23) | 9.81 (21.36) | 20.41 (24.73) | 0.265 |
| *E. casseliflavus* | 2.33 (7.29) | 2.36 (7.44) | 0.36 (1.03) | 0.57 (1.46) | 2.98 (8.61) | 0.639 |
| *E. faecalis* | 8.80 (17.07) | 9.48 (18.21) | 1.76 (3.98) | 4.95 (16.87) | 6.56 (14.60) | 0.854 |
| *E. faecium* | 7.49 (18.70) | 7.50 (18.89) | 0.77 (2.72) | 3.63 (13.02) | 9.00 (20.81) | 0.817 |
| *Escherichia* | 2.14 (3.81) | 1.99 (3.45) | 10.63 (18.96) | 11.02 (21.66) | 5.64 (8.92) | 0.099 |
| *E. coli* | 2.10 (3.69) | 1.94 (3.41) | 11.81 (21.93) | 10.74 (21.16) | 5.52 (8.77) | 0.101 |
| *Finegoldia* | 1.05 (3.16) | 0.95 (2.72) | 0.59 (1.58) | 0.63 (1.52) | 1.83 (5.60) | 0.854 |
| *F. magna* | 1.05 (3.09) | 0.95 (2.72) | 0.30 (0.97) | 0.63 (1.52) | 1.73 (5.60) | 0.854 |
| *Fusobacterium* | 1.83 (2.14) | 1.66 (1.94) | 2.95 (3.92) | 3.03 (3.56) | 1.11 (1.53) | 0.181 |
| *F. nucleatum* | 1.63 (2.04) | 1.49 (1.89) | 2.44 (3.71) | 2.76 (3.56) | 1.01 (1.53) | 0.301 |
| *Haemophilus* | 0.62 (0.90) | 0.62 (0.90) | 3.50 (7.51) | 3.82 (8.74) | 0.89 (1.47) | 0.128 |
| *H. parainfluenzae* | 0.48 (0.73) | 0.48 (0.72) | 2.51 (5.92) | 2.79 (7.08) | 0.68 (1.20) | 0.222 |
| *Hafnia* | 1.06 (3.04) | 0.96 (2.75) | 2.25 (5.76) | 2.76 (6.63) | 0.44 (1.28) | 0.491 |
| *H. alvei* | 1.06 (2.97) | 0.96 (2.75) | 0.50 (1.42) | 2.76 (6.63) | 0.44 (1.28) | 0.491 |
| *Klebsiella* | 8.95 (13.22) | 9.71 (14.35) | 17.81 (21.47) | 21.22 (23.02) | 9.80 (14.34) | 0.146 |
| *K. oxytoca* | 4.18 (7.87) | 4.24 (8.15) | 6.24 (9.97) | 9.22 (11.07) | 5.31 (9.59) | 0.479 |
| *K. pneumoniae* | 3.17 (5.44) | 3.58 (6.03) | 6.82 (14.62) | 9.59 (16.28) | 3.06 (5.83) | 0.198 |
| *K. variicola* | 1.42 (3.43) | 1.70 (4.19) | 1.58 (2.89) | 2.06 (3.87) | 1.24 (2.82) | 0.969 |
| *Lactobacillus* | 4.08 (8.73) | 3.99 (8.39) | 0.17 (0.29) | 0.24 (0.40) | 4.21 (8.86) | 0.177 |
| *L. animalis* | 1.78 (7.62) | 1.73 (7.37) | 0.01 (0.02) | 0.10 (0.34) | 2.03 (8.23) | 0.785 |
| *Nocardia* | 0.85 (1.77) | 0.08 (0.17) | 1.11 (1.39) | 0.03 (0.05) | 0.86 (1.34) | 0.021 |
| *N. brevicatena* | 0.78 (1.75) | 0.00 (0.01) | 0.76 (1.15) | 0.00 (0.00) | 0.78 (1.37) | 0.020 |
| *Parvimonas* | 1.41 (2.67) | 1.31 (2.41) | 0.87 (2.11) | 0.83 (1.88) | 0.94 (1.91) | 0.892 |
| *P. micra* | 1.41 (2.61) | 1.31 (2.41) | 0.75 (2.11) | 0.83 (1.88) | 0.89 (1.91) | 0.892 |
| *Prevotella* | 0.56 (1.35) | 0.51 (1.23) | 3.02 (7.74) | 3.46 (8.33) | 0.38 (0.59) | 0.176 |
| *P. denticola* | 0.27 (1.05) | 0.26 (0.98) | 1.78 (6.71) | 2.18 (7.43) | 0.11 (0.29) | 0.49 |
| *Raoultella* | 0.31 (1.12) | 0.31 (1.11) | 0.95 (3.45) | 1.36 (4.46) | 0.72 (2.67) | 0.778 |
| *R. ornithinolytica* | 0.31 (1.10) | 0.31 (1.11) | 0.95 (3.45) | 1.36 (4.46) | 0.72 (2.67) | 0.778 |
| *Serratia* | 0.20 (0.43) | 0.25 (0.58) | 1.19 (4.25) | 1.53 (4.82) | 0.32 (0.66) | 0.507 |
| *S. marcescens* | 0.16 (0.42) | 0.22 (0.57) | 1.15 (4.21) | 1.46 (4.78) | 0.30 (0.65) | 0.518 |
| *Shigella* | 0.23 (0.39) | 0.21 (0.39) | 1.26 (2.28) | 0.93 (1.96) | 0.65 (1.12) | 0.115 |
| *S. flexneri* | 0.18 (0.30) | 0.17 (0.31) | 1.04 (1.92) | 0.72 (1.55) | 0.53 (0.92) | 0.119 |
| *Streptococcus* | 27.93 (24.04) | 28.54 (24.39) | 5.13 (7.62) | 4.39 (4.42) | 22.22 (19.12) | 2.89E-05 |
| *S. anginosus* | 18.34 (18.38) | 19.03 (19.32) | 1.60 (2.15) | 2.97 (3.68) | 15.30 (15.67) | 0.0002 |
| *S. constellatus* | 3.41 (6.99) | 3.46 (7.18) | 0.74 (2.33) | 0.52 (0.88) | 3.75 (6.96) | 0.262 |
| *S. intermedius* | 1.55 (2.06) | 1.57 (2.12) | 0.42 (0.99) | 0.43 (0.91) | 1.35 (1.79) | 0.083 |
| *S. oralis* | 2.35 (8.59) | 2.32 (8.68) | 1.32 (5.51) | 0.07 (0.06) | 0.25 (0.27) | 0.713 |
| *Veillonella* | 4.98 (6.47) | 4.93 (6.15) | 11.92 (13.92) | 8.27 (9.26) | 3.70 (4.01) | 0.033 |
| *V. dispar* | 0.61 (1.72) | 0.65 (1.73) | 2.37 (7.84) | 0.54 (0.65) | 0.21 (0.21) | 0.441 |
| *V. parvula* | 4.36 (6.08) | 4.27 (5.89) | 8.56 (12.47) | 7.70 (8.85) | 3.48 (3.86) | 0.106 |
| *Yersinia* | 1.18 (4.93) | 1.16 (4.84) | 3.93 (15.88) | 4.86 (17.82) | 1.64 (6.42) | 0.799 |
| *Y. enterocolitica* | 1.03 (4.54) | 1.04 (4.54) | 3.54 (14.92) | 4.49 (16.75) | 1.48 (6.02) | 0.808 |
| Other | 17.00 (9.17) | 16.68 (9.57) | 15.30 (10.47) | 14.25 (8.68) | 16.36 (10.50) | 0.928 |
